# Analysis of the effect of the mitochondrial prohibitin complex, a context-dependent modulator of longevity, on the *C. elegans* metabolome^☆^

**DOI:** 10.1016/j.bbabio.2015.06.003

**Published:** 2015-11

**Authors:** Artur B. Lourenço, Celia Muñoz-Jiménez, Mónica Venegas-Calerón, Marta Artal-Sanz

**Affiliations:** a Andalusian Centre for Developmental Biology (CABD), CSIC, Universidad Pablo de Olavide-Junta de Andalucía, Carretera de Utrera, km 1, 41013 Sevilla, Spain; b Department of Biochemistry and Molecular Biology of Plant Products, Instituto de la Grasa (IG-CSIC), Ctra. Utrera Km 1, Campus Universitario Pablo de Olavide, 41013, Sevilla, Spain

**Keywords:** Prohibitin, Insulin signalling, Mitochondria, *C. elegans*, Longevity, Metabolomics

## Abstract

The mitochondrial prohibitin complex, composed of two proteins, PHB-1 and PHB-2, is a context-dependent modulator of longevity. Specifically, prohibitin deficiency shortens the lifespan of otherwise wild type worms, while it dramatically extends the lifespan under compromised metabolic conditions. This extremely intriguingly phenotype has been linked to alterations in mitochondrial function and in fat metabolism. However, the true function of the mitochondrial prohibitin complex remains elusive. Here, we used gas chromatography coupled to a flame ionization detector (GC/FID) and ^1^H NMR spectroscopy to gain molecular insights into the effect of prohibitin depletion on the *Caenorhabditis elegans* metabolome. We analysed the effect of prohibitin deficiency in two different developmental stages and under two different conditions, which result in opposing longevity phenotypes, namely wild type worms and *daf-2(e1370)* insulin signalling deficient mutants. Prohibitin depletion was shown to alter the fatty acid (GC/FID) and ^1^H NMR metabolic profiles of wild type animals both at the fourth larval stage of development (L4) and at the young adult (YA) stage, while being more pronounced at the later stage. Furthermore, wild type and the diapause mutant *daf-2(e1370)*, either expressing or not prohibitin, were clearly distinguishable based on their metabolic profiles, revealing changes in fatty acid composition, as well as in carbohydrate and amino acid metabolism. Moreover, the metabolic data indicate that *daf-2(e1370)* mutants are more robust than the wild type animals to changes induced by prohibitin depletion. The impact of prohibitin depletion on the *C. elegans* metabolome will be discussed herein in the scope of its effect on longevity. This article is part of a Special Issue entitled: Mitochondrial Dysfunction in Aging. Guest Editor: Aleksandra Trifunovic

## Highlights

• Impact of the mitochondrial prohibitin (PHB) complex on the *C. elegans* metabolome
• Depletion of individual PHB subunits results in similar metabolic profiles.
• PHB affects fatty acid composition, amino acid and carbohydrate metabolism.
• *daf-2* mutants are more robust than wild type worms to the effect of PHB depletion.
• Modulation of fermentation may contribute to the longevity of PHB-depleted worms.

## 1 Introduction

The nematode *Caenorhabditis elegans* has been extensively used to gain insights into the complex process of ageing and it has been extremely useful in the identification of mutations leading to increased longevity [1,2]. Research has led to remarkable progress in describing the molecular pathways that modulate ageing, including several signalling cascades, among them, the most prominent one being the insulin/insulin-like signalling (IIS) pathway [3]. One universal hallmark of ageing is the marked alterations in cellular energy metabolism [4]. Consistently, the biogenesis and function of mitochondria, the energy-generating organelles in eukaryotic cells, are primary longevity determinants [5]. Recently, the mitochondrial prohibitin complex was shown to differentially modulate longevity depending on intrinsic and extrinsic cues [6].

The mitochondrial prohibitin complex is composed of two proteins, PHB-1 and PHB-2, which bind to each other to form a heterodimer that is assembled into a ring-like macromolecular structure at the inner mitochondrial membrane [7]. These two subunits are interdependent for the formation of the complex, leading the absence of one of them to the absence of the whole complex [8–10]. Although different cellular functions have been suggested for both PHB-1 and PHB-2 in other cellular compartments, several evidences points to prohibitins functioning together within mitochondria (reviewed in [11,12]). Several roles have been proposed for the mitochondrial prohibitin complex, including a role as a membrane-bound chaperone, which holds and stabilizes newly synthesised mitochondrial-encoded proteins [13,14] and as scaffold proteins that recruit membrane proteins to a specific lipid environment [15,16]. Prohibitins have also been ascribed to different cellular processes such as cellular proliferation, cancer and ageing (reviewed in [12]). Prohibitin depletion delays development, reduces body size and gives rise to a wide range of somatic and germline defects, spanning from complete sterility to severely reduce brood sizes and a morphologically abnormal somatic gonad [6,8]. Intriguingly, prohibitin deficiency results in opposite ageing phenotypes depending on the metabolic status, being this phenotype evolutionarily conserved [6,17]. In particular, prohibitin deficiency shortens the lifespan of otherwise wild type nematodes, while it dramatically extends the lifespan of the already long-lived *daf-2(e1370)* insulin receptor mutants [6].

DAF-2, an orthologue of the insulin/insulin-like growth factor receptor, modulates the activity of the IIS pathway, through the FOXO transcription factor DAF-16 [18,19]. DAF-16 is required for reproductive growth and metabolism, as well as normal lifespan, responding to environmental cues, such as nutritional status or growth conditions [20,21]. Moreover, it regulates the entrance and exit into an alternative developmental stage, the diapause stage, characterised by extremely high resistance and survival to harsh conditions [20]. The diapause mutant *daf-2* is characterised by alterations in its metabolism, namely at the level of the glyoxylate shunt and gluconeogenesis [22,23]. Indeed, one plausible mechanism for the increased longevity of *daf-2* mutants involves its tuning of cellular metabolism towards maximal survival [23]. Nevertheless, how these metabolic shifts towards maximal survival occur, and how, if at all, they contribute, remains largely unknown. Interestingly, the modulation of lifespan by prohibitin is accompanied by alterations in the levels of fat content, as measured by different staining methods [6]. Additionally, prohibitin deficiency extends the lifespan of *nhr-49* mutant. NHR-49 is a key regulator of fat mobilization, modulating fat consumption and maintaining a normal balance of fatty acid composition [6]. In *C. elegans* up to 35% of the dry body mass is composed by lipids, from which 40–55%, depending on the diet and growth stage, are the fat stores triglycerides, esters derived from glycerol and three fatty acids [24,25]. Fat visualization using dyes, apart from the controversy on what these dyes are really staining [26], does not allow the distinction of the different lipid composition. Thus, a more direct way of examining the lipid composition upon prohibitin depletion and, in a broader perspective, a global view of the effect of prohibitin on the *C. elegans* metabolome is missing.

In recent years, the advances of different analytical platforms have allowed the development of the field of metabolomics. Metabolomics focus on the analysis of all the metabolites, the metabolome, of biological systems. However, the physicochemical diversity of the metabolites makes virtually impossible, even with all the advances made so far, to consider them all at the same time with a single strategy [27]. Instead, just to get near to its ultimate goal, a combination of different analytical methods and different metabolite extraction procedures must be used [27]. Application of a multitude and complementary metabolomics approaches will allow the gathering of molecular data that ultimately will led to a better understanding of the ageing process.

We present herein the first broad characterisation of the metabolic modifications induced by prohibitin depletion both in wild type and in *daf-2(e1370)* mutants. For that purpose, analysis of the fatty acid composition using gas chromatography coupled to a flame ionization detector (GC/FID) and ^1^H NMR metabolic profiling of whole worms was conducted. Prohibitin deficiency was found to induce a broad reorganization of the metabolic network in both wild type and *daf-2(e1370)* mutants, and these findings will be discussed in the scope of the observed effect of prohibitin on the worm lifespan expectancy.

## 2 Material and methods

### 2.1 Strains, worm culture and sampling conditions

Nematodes were cultured and maintained according to standard methods at 20 °C. The strains N2, wild type Bristol isolate, and CB1370, *daf-2(e1370)III*, were used in this study. Synchronous animal populations were generated by hypochlorite treatment of gravid adults. Tightly synchronised embryos were obtained by allowing eggs to hatch and develop until L1 larval stage in S Basal during overnight incubation. The worms were then fed with HT115 (DE3) *Escherichia coli* bacteria (deficient for RNase-E), harboring the appropriate RNAi plasmids (pL4440 for control RNAi, pL4440 containing a *phb-1* or a *phb-2* genomic fragment for RNAi knockdown of either *phb-1* or *phb-2*, respectively). Each bacterial strain was inoculated, from an overnight pre-inoculum, in LB (carbenicillin (25 mg/l) (Sigma) and tetracycline (15 mg/l) (Sigma)) to an initial OD_600 nm_ of approximately 0.2 and grown until it reached an OD_600 nm_ of approximately 1.5 (37 °C). IPTG (Sigma) was then added to the bacterial culture (1 mM). The bacterial culture was incubated for 2 h (37 °C) and then harvested by centrifugation at 3200 ×*g* (4 °C) for 20 min. Pellets were washed with S Basal (4 °C) and harvested again (3200 ×*g*, 20 min and 4 °C). Finally, bacterial stocks were prepared by resuspending the pellets (30 g/l) in S Medium containing carbenicillin (25 mg/l), IPTG (1 mM) and cholesterol (5 mg/l) (Sigma), and kept at 4 °C at most 4 days before being used. The effectiveness of RNAi against *phb-1* or *phb-2* was checked phenotypically by the worm sterility (data not shown). To assess the effect of either *phb-1* or *phb-2* deficiency on the whole worm GC/FID and ^1^H NMR profiles, synchronous populations of N2 wild type worms (approximately 2 worms/μl) were grown in liquid medium and sampled (approximately 30,000 worms) at either L4 larval or young adult (YA) stages. To compare the effect of prohibitin deficiency on the GC/FID and ^1^H NMR profiles of wild type and *daf-2(e1370)* worms, synchronous populations were grown on agar plates seeded with the appropriate bacteria (control RNAi or *phb-1(RNAi)*) and sampled (approximately 10,000 worms) at the YA stage. Sampled worms were washed six times (three with S Basal, two more with double distilled water and a sixth time with MS-grade water (Fluka)), harvested after each washing step by centrifugation at 800 ×*g* for 1 min (20 °C) and the final worm pellet was snap-frozen in liquid nitrogen and kept in the freezer (− 80 °C) until further use.

### 2.2 GC/FID analysis

To determine the overall fatty acid composition of the nematodes, lipids were extracted using an adaptation of the methyl-tert-butyl ether lipid extraction procedure [28]. Briefly, 1.5 ml of cold (− 25 °C) MS-grade methanol (Sigma) was added to the frozen worm pellet (200 μl) and transfer to a 10 ml glass vial. Worms were broken up by sonication (SONOPLUS 2070; 20 cycles of 1 min at maximum power followed by a pause of 15 s to make sure the mixture was maintained cold) in a cold ethanol bath (between − 20 °C and − 30 °C). After, 5 ml of methyl tertiary-butyl ether (MTBE) (Sigma) was added to this mixture, vortexed slightly and incubated for 1 h at 20 °C in a shaker. Phase separation was induced by adding 1.25 ml of MS-grade water (Fluka), vortexed slightly and, upon 10 min of incubation (20 °C), centrifuged at 1000 ×*g* for 10 min (20 °C). The upper phase (organic phase) was collected, while the lower phase was re-extracted with 2 ml of MTBE/methanol/water (10:3:2.5, v/v/v). Both organic phases were then combined and kept in the freezer (− 25 °C) until further use.

After lipid extraction, each sample was derivatised to make it amenable to be analysed by gas chromatography coupled to a flame ionization detector (GC/FID). First, the sample was evaporated in nitrogen atmosphere until dryness. To release the fatty acids of complex lipids and methylate them, 1 ml of hydrogen chloride (HCl)-methanol (1.25 M HCl) (Sigma) was added and kept in a bath at 80 °C for 1 h. Two phases were then obtained by the addition of 3 ml of hexane (Sigma) and 1.5 ml of Na_2_SO_4_ (6.7% m/v) (Sigma). Finally, the fatty acid methyl esters (FAMEs) were obtained by transferring the upper phase to another tube, where it was evaporated in the same conditions as before. In the end, 150 μl of heptane (Sigma) was added, FAMEs were resuspended and transferred to a vial with an insert for the GC/FID analysis and injected to the GC. The samples were analysed in a gas chromatograph (Perkin Elmer Clarus 500) equipped with a 0.2 μm × 60 m × 0.25 mm fused silica capillary column, hydrogen at 45 ml min^− 1^ was employed as carrier gas and coupled to a flame ionization detector. The gas chromatograph was programmed for an initial temperature of 140 °C (hold for 2 min), followed by an increase of 10 °C min^− 1^ to 210 °C (hold for 7 min). Also set were the following parameters: 270 °C inlet and 280 °C detector temperatures. To assign the peaks in the spectral data, a standard mixture with known composition of FAMEs (Sigma) was previously run in the same conditions, and the assignment was done based on the retention time and the area of the peaks.

### 2.3 ^1^H NMR metabolic profiling

To obtain the metabolic profile of whole worm extracts, metabolites were extracted using an adaption of hot ethanol extraction procedure [29]. In short, 1.0 ml of cold (− 25 °C) MS-grade ethanol (Merck) was added to the worm pellet (200 μl) and transferred to a 10 ml glass vial. Worms were broken up by sonication using the same procedure described before for the GC/FID analysis. The broken worms were centrifuged for 3 min at 3200 ×*g* in a centrifuge previously cooled to − 10 °C and the supernatant was kept on cold. Hot ethanol (80 °C) was added to the pellet (0.75 ml of 75% ethanol (v/v)), vortexed for 15 s, incubated for 3 min at 80 °C, and vortexed again for 15 s. The mixture was centrifuged again for 3 min at 3200 ×*g*. This process was done two times and the three supernatants were joined together. The samples were concentrated under vacuum and kept in the freezer (− 80 °C) until NMR data acquisition.

The samples were analysed using a 600 MHz Bruker spectrometer, equipped with a 5-mm cryogenically-cooled TCI probe (^1^H, ^15^N, ^13^C), at 298.15 K. Briefly, the dried extracts were dissolved in 0.7 ml of buffer (0.1 M phosphate buffer pH 7.00, in D_2_O, containing 0.1 mM of trimethylsilyl-2,2,3,3-tetradeuteropropionate (d4-TSP)), centrifuged at 16,000 ×*g* for 10 min and the supernatant was transferred into 5 mm diameter NMR tubes. One-dimension (1D) proton spectra were acquired at constant gain, with 256 transients and a spectral width of 20 ppm. A train of Carr–Purcell–Meiboom–Gill (CPMG) echoes, of 40 ms long, was used to reduce macromolecules broad signals. During the 4 s relaxation delay, the residual water peak was pre-saturated. All proton spectra were manually phased, baseline corrected and referenced to d4-TSP (δ 0.00 ppm) using the program Mnova v9 for NMR data processing. To aid in metabolite identification, 2D *J*-resolved (relaxation delay of 2 s, 64 increments and 8 transients), Total Correlation Spectroscopy (TOCSY; relaxation delay of 1.5 s, 256 increments and 16 transients) and Heteronuclear Single Quantum Coherence (HSQC; relaxation delay of 1.5 s, 360 increments and 88 transients) spectra were collected for representative samples.

### 2.4 Data analysis

For each GC/FID spectrum, the areas of the assigned peaks were calculated and normalised to their total area. All spectra were put in a data table form and imported into the software SIMCA v13.0.3.0 for multivariate data analysis. Regarding the ^1^H NMR data, the regions corresponding to residual water and ethanol (5.00–4.665, 3.70–3.63 and 1.21–1.15 ppm) were excluded. Each spectrum was integrated over a series of fixed width bins (0.005 ppm) using Mnova v9 features. All proton spectra were normalised to the total intensity between 9.70 and 0.70 ppm, put in a data table form and imported into the software SIMCA v13.0.3.0 for multivariate data analysis.

In SIMCA v13.0.3.0 both GC/FID and ^1^H NMR data were pre-processed using Pareto scaling, and analysed using principal component analysis (PCA) and orthogonal partial least squares discriminant analysis (O-PLS-DA). PCA, an unsupervised method, was used to get an overview of the data structure, to observe clustering or separation trends. R^2^X and Q^2^ (calculated by 7-fold internal cross-validation) values were considered as measures of goodness of model and the model robustness, respectively. R^2^X is the total variance explained by the set of principal components (PCs) of the model, and cross validation of each component will provide the Q^2^, which describes the total variation predicted by the model's PCs. The value of Q^2^ ranges from 0 to 1 and typically a Q^2^ value greater than 0.4 is considered a good model, and those with Q^2^ values over 0.7 are robust. O-PLS-DA, a supervised method that uses prior information, was used to better discriminate between clusters. Evaluation of the supervised models were based on the R^2^X, Q^2^, and also using the goodness-of-fit parameter R^2^Y (variation in class membership explained by the model). In the O-PLS-DA, a measure of the degree to which a particular variable explains cluster membership was obtained with the variable importance in projection for independent variables (VIP) plot (with confidence intervals derived from jack knifing routine). A VIP value above 1 indicates that a given variable is important for class membership. Identification of metabolites responsible for the cluster separation (variables combining high model influence with high reliability) was also done using the predictive S-line plot (a way to visualize both the covariance and the correlation structure between the X-variables and the predictive score).

Univariate statistical analysis was performed in excel. The relative abundances of the assigned peaks in the GC/FID data and the relative abundances of the characteristic bins (bins more characteristic of each specific metabolite) in the NMR data were analysed using two-sample for mean Student's t-test (unpaired, two tails and unequal variances). P-values of < 0.05 were considered to be statistically significant.

## 3 Results

### 3.1 Prohibitin depletion alters the fatty acid composition of wild type worms

**Fig. 1:**
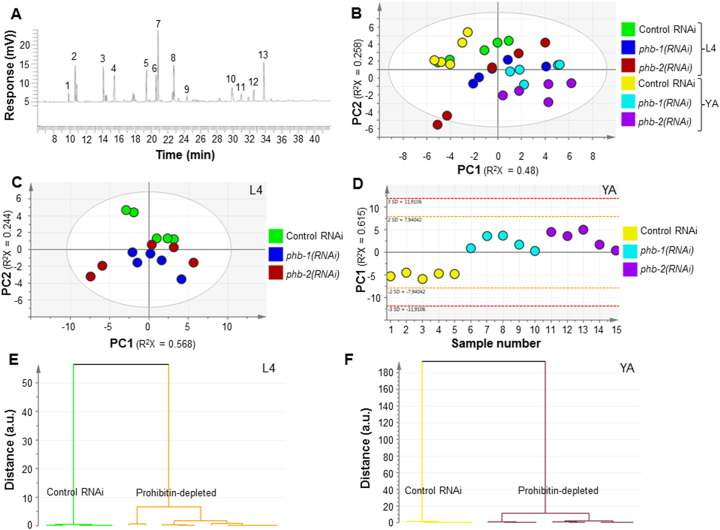
Multivariate analysis of the fatty acid composition of wild type worms upon prohibitin depletion. (A) Typical GC/FID spectrum of whole worm extracts. (B) Score plot derived from the PCA modelling (5 PCs, R^2^X = 0.948 and Q^2^ = 0.616) of the GC/FID data of wild type animals depleted of either *phb-1* or *phb-2* (control RNAi, *phb-1(RNAi)* and *phb-2(RNAi)*) at two developmental stages (fourth larval stage (L4) and young adults (YA)). (C and D) Score plots of the PCA model at the L4 stage (2 PCs, R^2^X = 0.812 and Q^2^ = 0.579) and at YA stage (1 PC, R^2^X = 0.615 and Q^2^ = 0.433), respectively. (E and F) Dendrograms derived from the O-PLS-DA modelling of GC/FID data at the L4 stage (1 + 2 + 0 PCs, R^2^X = 0.886, R^2^Y = 0.852 and Q^2^ = 0.741) and at the YA stage (1 + 1 + 0 PCs, R^2^X = 0.743, R^2^Y = 0.929 and Q^2^ = 0.89) respectively.

**Fig. 2:**
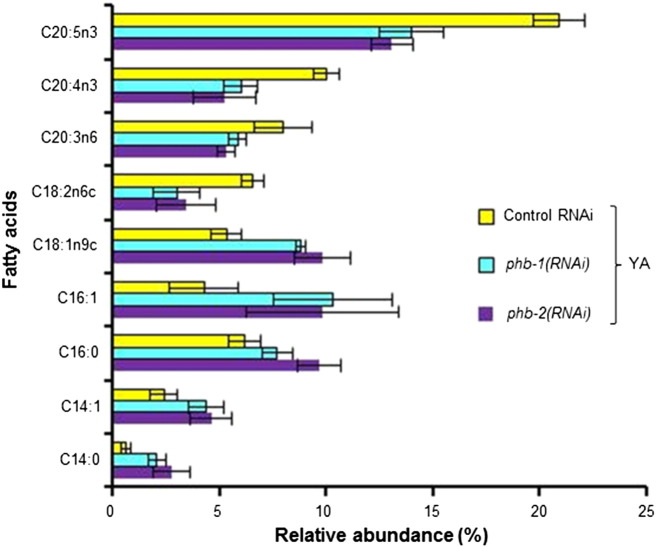
Fatty acid composition of wild type worms upon prohibitin depletion. Fatty acids showing an altered content at the young adult stage in a prohibitin-dependent manner are depicted (t-test, P-value < 0.05).

Prohibitin depletion strongly alters *C. elegans* fat content as indicated by a variety of both fixed and live staining methods [6]. Here, we initially asked if the mitochondrial prohibitin complex modulates the worm fatty acid composition by analysing the effect of depleting either *phb-1* or *phb-2* by RNA interference (RNAi). We analysed an organic fraction of the metabolome of wild type animals depleted of either prohibitin subunit at two developmental stages, the fourth larval stage of development (L4) and young adults (YA). The GC/FID analysis from whole worm extracts enabled the detection of several peaks, 13 of which were assigned and included in the multivariate analysis (Fig. 1; Table S1). Principal component analysis (PCA) evidences that along development the fatty acid composition changes in a prohibitin-dependent manner (Fig. 1B). Prohibitin-depleted worms (*phb-1(RNAi)* and *phb-2(RNAi)*) at the young adult stage appear more distant apart from wild type worms (control RNAi) than at the fourth larval stage of development (Fig. 1B). PCA modelling of either L4 or YA data sets separately makes this finding clearer (Fig. 1C and D). At the L4 stage, although the separation is still not completely clear, it is visible a distinction between wild type and prohibitin-depleted worms across principal component 2 (PC2) (Fig. 1C). Remarkably, the difference between wild type and prohibitin-depleted worms is more evident at the YA stage, clearly separated across PC1, while no clear separation is visible between *phb-1* or *phb-2* depleted worms (Fig. 1D). The dendrograms derived from the supervised models, using orthogonal partial least squares discriminant analysis (O-PLS-DA), clearly show that the two clusters (wild type and prohibitin-depleted worms) are more distant apart at the YA than at the L4 stage, as shown by the distance in the dendrograms (Fig. 1E and F). The supervised modelling at the YA stage pointed C20:5n3 and C20:4n3 as the fatty acids contributing more for cluster separation. The content of these two fatty acids is clearly lower in prohibitin deficient worms at the YA stage (Fig. 2; Table S1B). Besides these two fatty acids, C20:3n6 and C18:2n6c were also found to be reduced in prohibitin-depleted worms, while C18:1n9c, C16:1, C16:0, C14:1 and C14:0 were found to be increased upon prohibitin depletion (Fig. 2; Table S1B). Overall, RNAi targeting either *phb-1* or *phb-2* results in similar altered fatty acid composition, likely due to depletion of the whole PHB complex. In particular, prohibitin depletion in otherwise wild type animals leads to an increase in the content of short chain and saturated fatty acids, with the concomitant decrease of long chain and unsaturated fatty acids.

### 3.2 ^1^H NMR reveals clear changes in the metabolic profiles of wild type worms upon prohibitin depletion

**Fig. S1:**
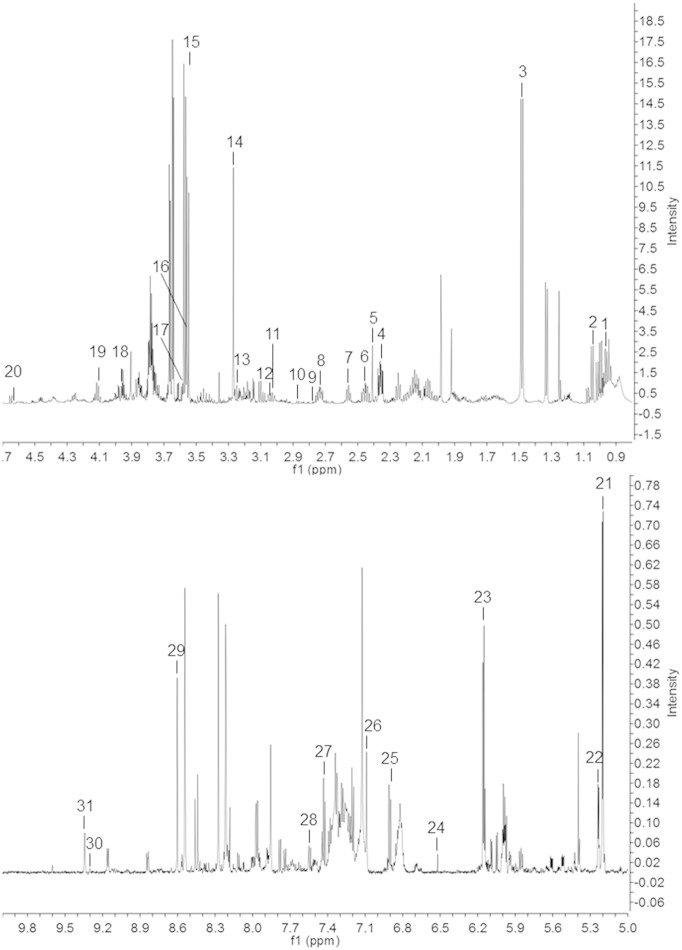
Typical ^1^H NMR spectrum of whole worm extracts. Key: 1) leucine; 2) valine; 3) alanine; 4) glutamate; 5) succinate; 6) glutamine; 7) beta-alanine; 8) cystathionine; 9) aspartate; 10) asparagine; 11) lysine; 12) ornithine; 13) arginine; 14) betaine; 15) glycerol; 16) glycine; 17) threonine; 18) serine; 19) lactate; 20) glutathione; 21) trehalose; 22) glucose; 23) ATP/ADP; 24) fumarate; 25) tyrosine; 26) histidine; 27) phenylalanine; 28) tryptophan; 29) AMP; 30) NADP^+^; 31) NAD^+^.

**Fig. 3:**
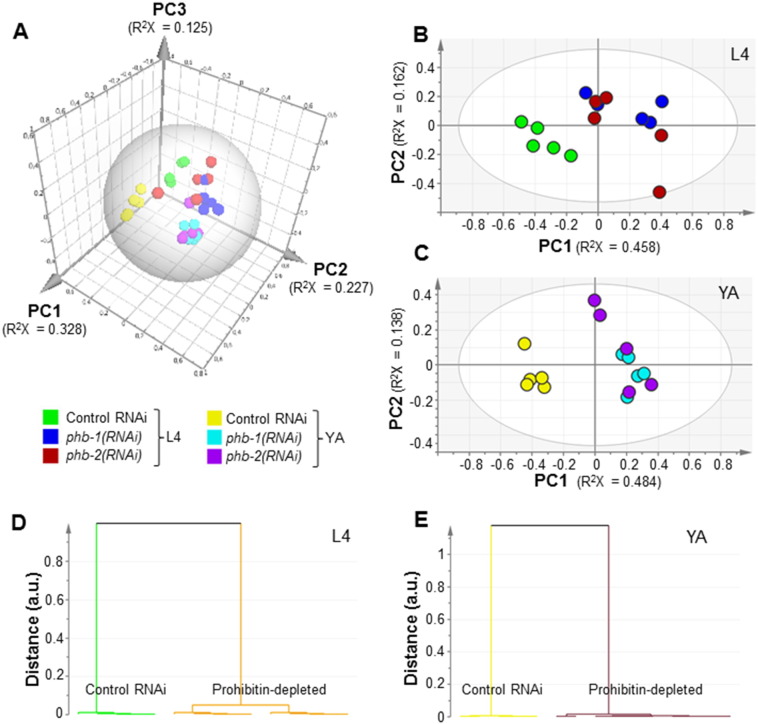
^1^H NMR metabolic profiling of wild type worms upon either *phb-1* or *phb-2* depletion. (A) 3D score plot derived from the PCA modelling (4 PCs, R^2^X = 0.732 and Q^2^ = 0.581) of the ^1^H NMR data (control RNAi, *phb-1(RNAi)*, *phb-2(RNAi)* at two developmental stages (fourth larval stage of development (L4) and young adult (YA) stages)). Score plots of the PCA model at L4 stage (B) (3 PCs, R^2^X = 0.726 and Q^2^ = 0.398) and at YA stage (C) (2 PCs, R^2^X = 0.622 and Q^2^ = 0.438). Dendrograms derived from the O-PLS-DA modelling of the ^1^H NMR data at L4 stage (D) (1 + 1 + 0 PCs, R^2^X = 0.605, R^2^Y = 0.926 and Q^2^ = 0.854) and at YA stage (E) (1 + 1 + 0 PCs, R^2^X = 0.619, R^2^Y = 0.98 and Q^2^ = 0.948).

**Fig. 4:**
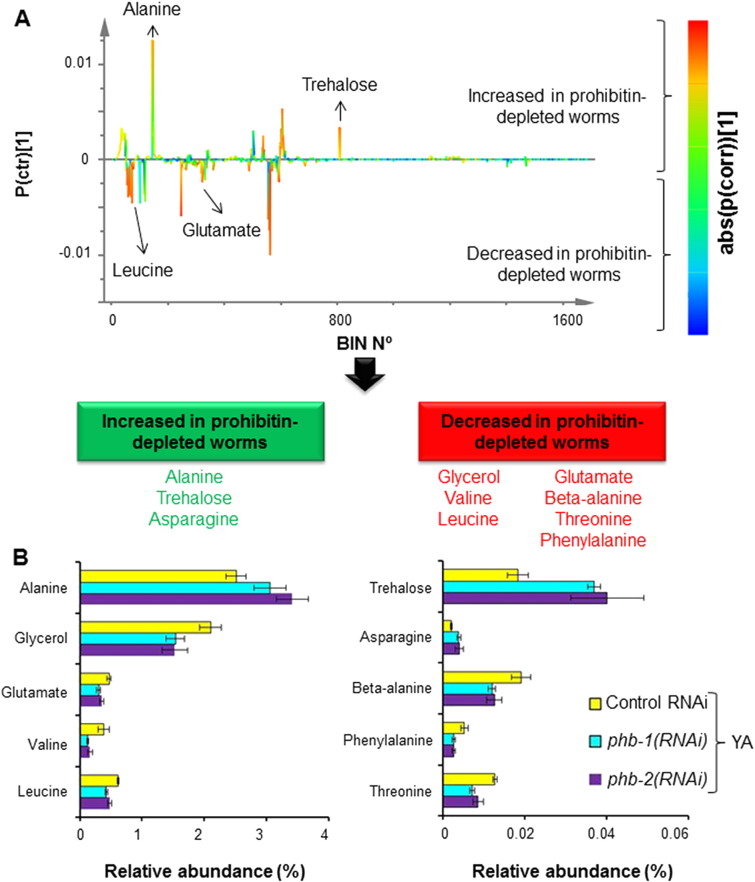
Metabolites showing an altered content in a prohibitin-dependent manner. (A) S-line plot from the O-PLS-DA modelling (1 + 1 + 0 PCs, R^2^X = 0.619, R^2^Y = 0.98 and Q^2^ = 0.948) of the ^1^H NMR data set from young adult wild type treated with RNAi targeting either *phb-1* or *phb-2*. (B) Relative abundances of the metabolites, based on a characteristic bin, displaying an altered content in a prohibitin-dependent manner (t-test, P-value < 0.05). Similar results were obtained for *phb-1/2(RNAi)* treated worms.

We then asked if the effect of prohibitin propagates more broadly over the worm metabolic network. To address this question, we undertook a metabolic profiling strategy by ^1^H NMR spectroscopy of whole worm extracts. Wild type worms, grown in liquid medium, either expressing (control RNAi) or not (*phb-1(RNAi)* or *phb-2(RNAi)*) prohibitin were sampled at two developmental stages (L4 and YA). Despite many resonances remained unassigned, a wide range of classes of biomolecules were identified, including amino acids (e.g. alanine and asparagine), cofactors (NAD^+^ and NADP^+^), sugars (e.g. glucose and trehalose) and organic acids (e.g. succinic and fumaric acids) (Fig. S1). Metabolite identification was done by a careful analysis of the chemical shifts, intensities, J couplings and multiplicities of the peaks present in the one dimensional ^1^H NMR spectra, together with information from J-resolved, TOCSY and HSQC spectra, and complemented with data gathered in different databases [30,31]. PCA of the NMR data evidences the existence of four main clusters (Fig. 3A). Namely, wild type worms at the L4 larval stage, wild type worms at the YA stage, prohibitin-depleted worms (*phb-1(RNAi)* and *phb-2(RNAi)*) at the L4 stage, and prohibitin-depleted worms at the YA stage (Fig. 3A). Similarly to the GC/FID data, PCA modelling of either L4 or YA data sets separately did not evidence any clear separation between worms depleted of either *phb-1* or *phb-2*, and evidence a clear distinction between wild type and prohibitin-depleted worms both at the L4 and YA stages (Fig. 3B and C). Moreover, the difference between wild type and prohibitin-depleted worms is higher at the YA than at the L4 stage, as shown by the distance between the clusters in the dendrograms derived from the supervised models (Fig. 3D and E). From the supervised model we were able to identify as the strongest contributors for cluster separation at the YA stage the metabolites alanine and trehalose, with an increase in their content, and glycerol and valine with a decrease in their content upon prohibitin depletion (Fig. 4A and B; Table S2). Altogether, the ^1^H NMR metabolic profiling of wild type worms revealed a broad metabolic reorganization, at level of both carbohydrate and amino acid metabolism, upon prohibitin depletion, which may partially contribute for the pleiotropic effect of prohibitin depletion in the physiology of *C. elegans*.

### 3.3 Prohibitin depletion has a more pronounced effect on the fatty acid composition of wild type worms than of *daf-2(e1370)* mutants

**Fig. S2:**
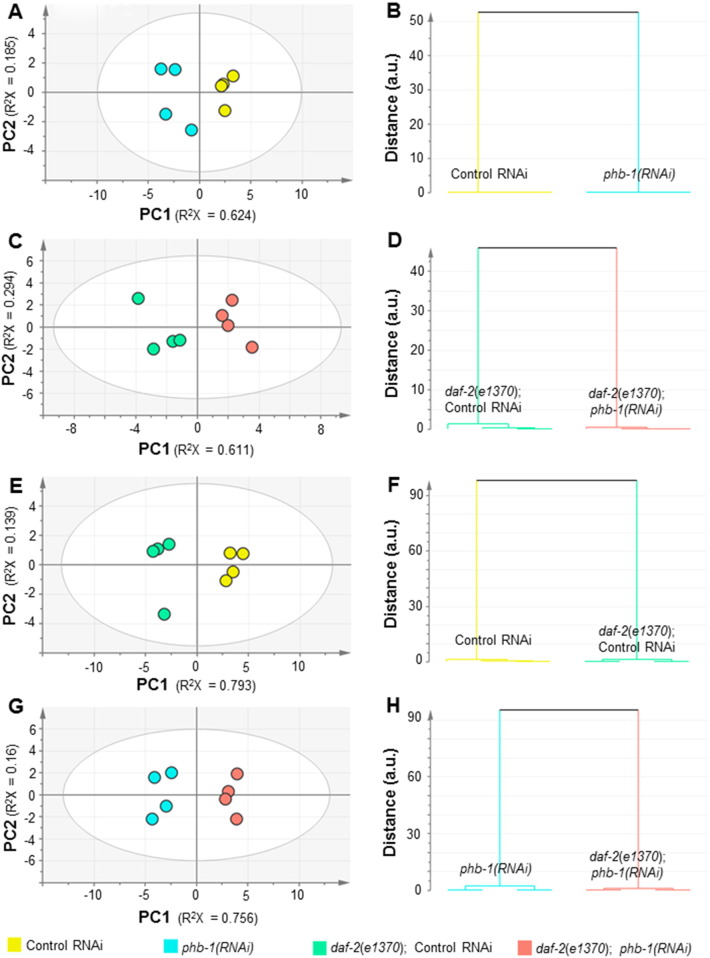
Multivariate analysis of the fatty acid composition in wild type and *daf-2(e1370)* worms upon prohibitin depletion. (A) PCA modelling (2 PCs, R^2^X = 0.809, Q^2^ = 0.401) and (B) O-PLS-DA modelling (1 + 3 + 0 PCs, R^2^X = 0.938, R^2^Y = 0.998 and Q^2^ = 0.971) for the comparison between control RNAi and *phb-1(RNAi)*. (C) PCA modelling (3 PCs, R^2^X = 0.969, Q^2^ = 0.818) and (D) O-PLS-DA modelling (1 + 1 + 0 PCs, R^2^X = 0.888, R^2^Y = 0.959 and Q^2^ = 0.928) for the comparison between *daf-2(e1370)*; control RNAi and *daf-2(e1370)*; *phb-1(RNAi)*. (E) PCA modelling (3 PCs, R^2^X = 0.984, Q^2^ = 0.920) and (F) O-PLS-DA modelling (1 + 0 + 0 PCs, R^2^X = 0.793, R^2^Y = 0.972 and Q^2^ = 0.957) for the comparison between control RNAi and *daf-2(e1370)*; control RNAi. (G) PCA modelling (2 PCs, R^2^X = 0.917, Q^2^ = 0.762) and (H) O-PLS-DA modelling (1 + 0 + 0 PCs, R^2^X = 0.756, R^2^Y = 0.969 and Q^2^ = 0.963) for the comparison between *phb-1(RNAi)* and *daf-2(e1370)*; *phb-1(RNAi)*.

**Fig. 5:**
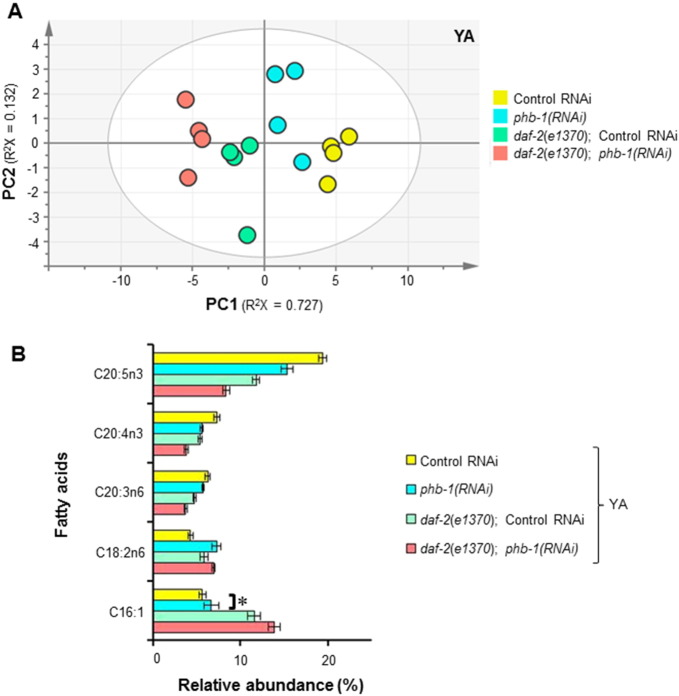
Prohibitin depletion alters the fatty acid composition of wild type and *daf-2(e1370)* worms in a similar fashion. (A) Score plot derived from the PCA model (3 PCs, R^2^X = 0.938 and Q^2^ = 0.772) of the GC/FID data (control RNAi, *phb-1(RNAi)*, *daf-2(e1370)*; control RNAi, *daf-2(e1370)*; *phb-1(RNAi)*). (B) Fatty acids with their content altered, in a similar fashion, upon prohibitin depletion both in wild type and *daf-2(e1370)* worms at YA stage (t-test, P-value < 0.05). The comparison signed by * is not statistically significant.

Prohibitin deficiency differentially modulates fat content in wild type and in *daf-2(e1370)* mutant animals [6]. To gain molecular insights associated with these differences, we inquire if prohibitin modulates fatty acid composition in a genetic background-specific manner. To achieve this, wild type and *daf-2(e1370)* worms either expressing (control RNAi) or not (*phb-1(RNAi)*) prohibitin were grown on agar plates and sampled at the YA stage. Prohibitin depletion in otherwise wild type worms results in a developmental delay, reaching the YA stage approximately 60 h after the L1 larval stage, as compared to 46 h on control RNAi. On the other hand, the *daf-2(e1370)* mutant takes 70 h to reach the YA stage on control RNAi, while on *phb-1(RNAi)* it takes 94 h. The conditions were set based on the previous results, which showed that RNAi targeting either *phb-1* or *phb-2* had a similar effect on the fatty acid composition of wild type worms, being these alterations more pronounced at the YA stage (Fig. 1). Finally, worms were grown on plate due to the higher frequency of *daf-2(e1370)* worms entering the dauer stage when grown in liquid medium, especially when feeding on *phb-1(RNAi)* bacteria, and more generally due to the difficulties to interpret metabolic and longevity analysis from worms subjected to the stresses present in liquid medium (reviewed in [32]). The GC/FID data indicate that depletion of prohibitin alters the fatty acid composition of both wild type and *daf-2(e1370)* worms grown on plate (Fig. 5 and Table S3). PCA modelling shows that wild type, *phb-1(RNAi)*, *daf-2(e1370)* and *daf-2(e1370)*; *phb-1(RNAi)* animals at the YA stage are clearly distinguishable across PC1 (Fig. 5A), indicating that fatty acid composition differs between wild type and *daf-2(e1370)* worms, and that in both cases it is altered, with the same general trend, upon prohibitin depletion. The effect of prohibitin depletion is more pronounced in wild type animals than in *daf-2(e1370)* mutant worms, as shown by the distance in the dendrograms derived from the supervised models (Fig. S2B and D). Nevertheless, the alteration in the fatty acid composition induced by prohibitin depletion in both genetic backgrounds is smaller than the differences between the two genetic backgrounds (Figs. 5A and S2). Compared to wild type worms, *daf-2(e1370)* mutants have a higher content of C14:0, C14:1, C16:1, and C18:2n6c and a lower content of C18:0, C20:0, C20:3n6, C20:4n6, C20:4n3 and C20:5n3 (t-test, P-value < 0.05) (Table S3). Most of the observed differences between wild type and *daf-2(e1370)* mutants are in agreement with previously published results [33], which reflect, a general increase in short chain and saturated fatty acids and a decrease in long chain and more unsaturated fatty acids. Prohibitin depletion in *daf-2(e1370)* mutants further increases the content of C16:1 and C18:2n6c while it further reduces the content of C20:0, C20:3n6, C20:4n3 and C20:5n3 (t-test, P-value < 0.05) (Fig. 5B; Table S3). Collectively, and similar to the phenotype observed in wild type worms, prohibitin depletion in *daf-2* mutants leads to a decrease in the content of long chain and unsaturated fatty acids, with the concomitant increase in the content of short chain and saturated fatty acids.

### 3.4 ^1^H NMR metabolic profiles reveal more dramatic changes in wild type worms than in *daf-2(e1370)* mutants upon prohibitin depletion

**Fig. S3:**
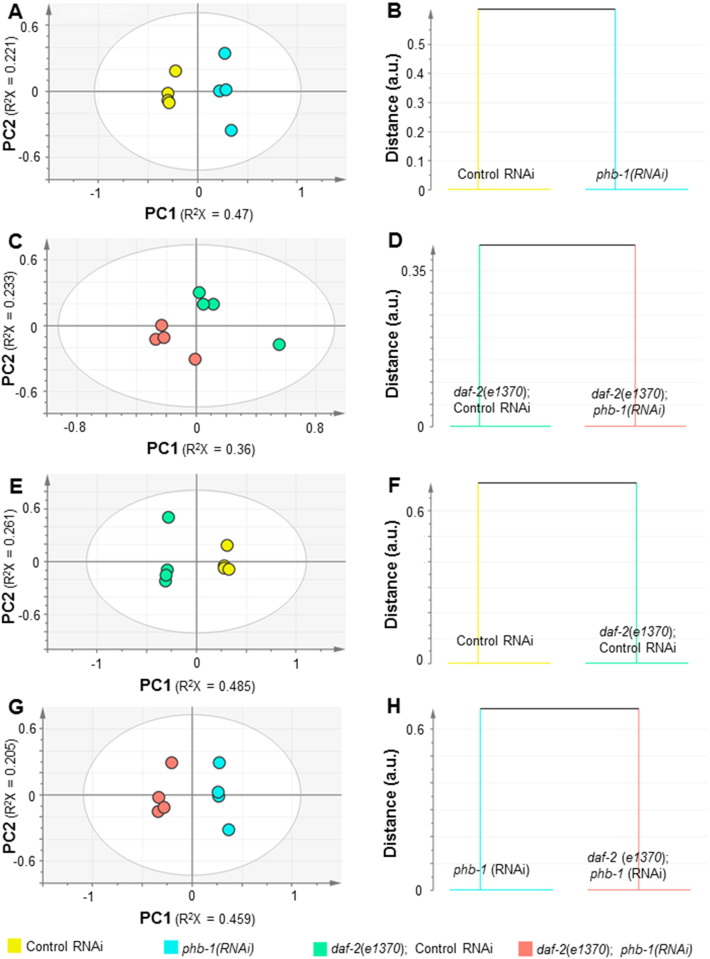
Multivariate analysis of the ^1^H NMR data from wild type and *daf-2(e1370)* worms upon prohibitin depletion. (A) PCA modelling (2 PCs, R^2^X = 0.691, Q^2^ = 0.308) and (B) O-PLS-DA modelling (1 + 1 + 0 PCs, R^2^X = 0.586, R^2^Y = 0.999 and Q^2^ = 0.975) for the comparison between control RNAi and *phb-1(RNAi)*. (C) PCA modelling (2 PCs, R^2^X = 0.593, Q^2^ = 0.0583) and (D) O-PLS-DA modelling (1 + 4 + 0 PCs, R^2^X = 0.873, R^2^Y = 1 and Q^2^ = 0.970) for the comparison between *daf-2(e1370)*; control RNAi and *daf-2(e1370)*; *phb-1(RNAi)*. (E) PCA modelling (2 PCs, R^2^X = 0.746, Q^2^ = 0.402) and (F) O-PLS-DA modelling (1 + 2 + 0 PCs, R^2^X = 0.796, R^2^Y = 1 and Q^2^ = 0.982) for the comparison between control RNAi and *daf-2(e1370)*; control RNAi. (G) PCA modelling (2 PCs, R^2^X = 0.664, Q^2^ = 0.342) and (H) O-PLS-DA modelling (1 + 1 + 0 PCs, R^2^X = 0.557, R^2^Y = 0.999 and Q^2^ = 0.943) for the comparison between *phb-1(RNAi)* and *daf-2(e1370)*; *phb-1(RNAi)*.

**Fig. 6:**
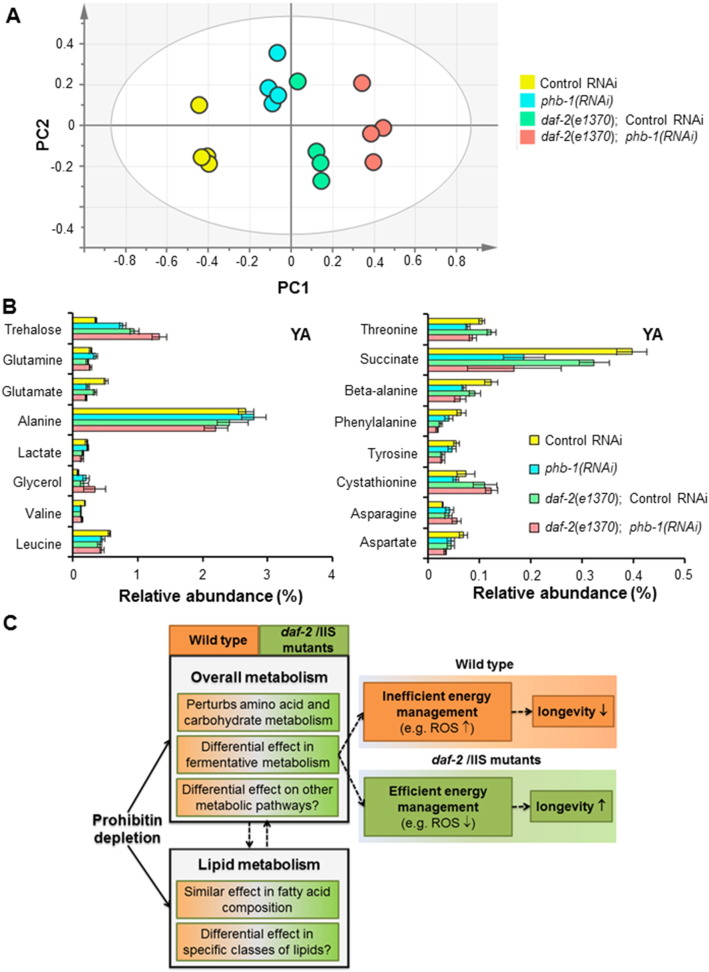
^1^H NMR metabolic profiling of wild type and *daf-2(e1370)* worms upon prohibitin-depletion. (A) Score plot derived from the PCA modelling (3 PCs, R^2^X = 0.674 and Q^2^ = 0.431) of the ^1^H NMR data (control RNAi, *phb-1(RNAi)*, *daf-2(e1370)*; control RNAi, *daf-2(e1370)*; *phb-1(RNAi)*. (B) Relative abundances of the metabolites, based on a characteristic bin, displaying an altered content upon prohibitin depletion. (C) Model on how changes on the worm metabolome upon prohibitin depletion might lead to opposing effects on longevity. See detailed explanation in the main text of the discussion. ROS — reactive oxygen species.

The broad alteration of the metabolic profile observed in wild type worms upon prohibitin depletion, prompted us to examine its effect on the ^1^H NMR profile of *daf-2* mutant animals. We analysed the changes in the metabolic profiles of wild type and *daf-2(e1370)* either expressing (control RNAi) or not (*phb-1(RNAi)*) prohibitin. Worms were grown on solid media and analysed at the YA stage. Prohibitin depletion alters the metabolic profiles of both wild type and *daf-2(e1370)* worms, as seen in the PCA model (Fig. 6A), which shows a clear separation of the four groups of samples across PC1. Prohibitin depletion induces smaller changes on the metabolic profile of *daf-2(e1370)* mutants than of wild type animals, as indicated by the distance in the dendrograms derived from the supervised models (Fig. S3B and D). Similar to what was observed on the fatty acid composition, the changes induced by prohibitin depletion are smaller than the differences on the metabolic profiles between the two genetic backgrounds (Figs. 6A and S3). The content of leucine, valine, glutamate, succinate, beta-alanine, aspartate, ornithine, threonine and phenylalanine was found to be lower, while the content of glutamine, asparagine, arginine, glycine, glycerol, trehalose and allantoin was found to be higher in prohibitin-depleted worms (t-test, P-value < 0.05) (Fig. 6B and Table S4). The comparison between wild type worms and *daf-2(e1370)* mutants fed on control RNAi showed that the later has higher content of betaine, threonine, trehalose and allantoin, while wild type animals have higher content of leucine, valine, lactate, glutamate, glutamine, succinate, beta-alanine, aspartate, lysine, ornithine, glycine, tyrosine, phenylalanine and tryptophan (t-test, P-value < 0.05) (Fig. 6B and Table S4). Most of these differences are in agreement with previously published data [33]. Additionally, and similar to its effect on wild type worms, prohibitin depletion in *daf-2(e1370)* mutants leads to a decrease in the content of glutamate, beta-alanine, threonine and phenylalanine, while it leads to an increase in the content of glutamine and trehalose (t-test, P-value < 0.05) (Fig. 6B and Table S4). On the other hand, depletion of prohibitin had an opposing effect in the content of alanine in the two genetic backgrounds, which lead to a significant difference between the two genetic backgrounds fed on *phb-1(RNAi)*. Although not statistically significant, wild type worms have higher content of alanine than *daf-2* mutants. However, the content of alanine is further increased upon prohibitin depletion in wild type worms and further decreased in *daf-2* mutants, which results in *daf-2(e1370)* mutants fed on *phb-1(RNAi)* having a significant lower content of alanine than prohibitin depleted otherwise wild type worms (Fig. 6B and Table S4). The alterations in the levels of alanine show an inverse trend with the longevity of the worms, which might be relevant when considering that the same inverse correlation between alanine content and yeast chronological lifespan has been previously reported [34]. A similar behaviour in the content of lactate was also observed (Fig. 6B and Table S4). Overall, prohibitin depletion had a more pronounced effect on the metabolic profile of wild type worms than of *daf-2(e1370)* mutants indicating that *daf-2* mutants are more robust to the changes elicited upon prohibitin depletion, and which may account to some extent for the opposing effect of prohibitin on the worms longevity.

## 4 Discussion

The mitochondrial prohibitin (PHB) complex is composed of about 12 to 16 heterodimers, each constituted by two subunits, PHB-1 and PHB-2. Both subunits are interdependent for the formation of the complex, leading the absence of one of them to the absence of the whole complex [8–10]. Different reports have argued that PHB-1 and PHB-2 can either function together within mitochondria, in the mitochondrial PHB complex, or have different functions in other cellular compartments (reviewed in [11,35]). However, the exact function of either PHB-1 or PHB-2 remains elusive. Herein, we show that depletion of either PHB-1 or PHB-2 by RNAi specifically targeting each of these subunits has a similar effect on both the fatty acid and ^1^H NMR metabolic profiles of otherwise wild type worms. Therefore, the results obtained herein argue in favour of PHB-1 and PHB-2 functioning together within the mitochondrial prohibitin complex, and highlight the potential of metabolomics as a functional genomics tool [36,37]. Additionally, we show that prohibitin affects the fatty acid and ^1^H NMR metabolic profiles of otherwise wild type worms more deeply at the young adult (YA) stage than at the fourth larval developmental (L4) stage. This indicates that the metabolic profiles change along development in a prohibitin-dependent manner. Since during ageing the content of fat is altered in a prohibitin-dependent manner [6], it is likely that prohibitin depletion leads to even more pronounced alterations on both the fatty acid and ^1^H NMR metabolic profiles of aged worms. Altogether, the GC/FID and ^1^H NMR data evidence a broad reorganization of the metabolic network upon prohibitin depletion in otherwise wild type worms.

The mitochondrial prohibitin complex was suggested to affect longevity by modulating mitochondrial function and fat metabolism in *C. elegans* in a genetic-background-specific manner [6]. These led us to hypothesize that the effect of prohibitin on the *C. elegans* metabolome might account for its impact in the ageing process. In agreement with previously published data [33,38], the fatty acid composition of *daf-2(e1370)* mutants is clearly different from wild type animals. Strikingly, prohibitin deficiency has an additive effect, although less pronounced, on many of the changes observed in the loss-of-function mutant *daf-2(e1370)*. In particular, prohibitin deficiency leads to changes in the content of polyunsaturated fatty acids (PUFAs), which have been linked to longevity [38,39]. It was previously shown that the fatty acid chain length and susceptibility to oxidation decreased sharply in long‐lived mutants of the insulin like‐signalling pathway, correlating extremely well with the lifespan of these worms [38]. In agreement with this observation, the increase in the lifespan from wild type to *daf-2(e1370)*, and from the later to prohibitin-depleted *daf-2(e1370)* worms [6] is accompanied by changes in the fatty acid composition that overall followed this trend, particularly a decrease in the content of long chain and more unsaturated fatty acids. Intriguingly, the fatty acid composition of prohibitin-depleted wild type worms, which are short-lived [6], is altered in a similar fashion, having prohibitin depletion a higher impact in the fatty acid composition of wild type worms than of *daf-2(e1370)* mutants. Therefore, the changes in the fatty acid composition at the YA stage cannot, by their own, account for the opposing effect of prohibitin on longevity. However, it is reasonable to speculate that those changes coupled to alterations in energy metabolism might have implications in lifespan determination. Interestingly, it was found that prohibitin deficiency extends the lifespan of both *nhr-49* and *fat-7* mutants [6]. The nuclear hormone receptor NHR-49 regulates fat mobilization by regulating the expression of a wide variety of genes, including mitochondrial fatty acid β-oxidation enzymes and Δ9 stearoyl-CoA desaturases, such as FAT-7, involved in the synthesis of monounsaturated fatty acids [40].

Prohibitin deficiency also has a more pronounced effect on the amino acid and carbohydrate metabolism of otherwise wild type animals than of *daf-2(e1370)* mutants, as assessed by ^1^H NMR metabolic profiling. The supplementation of different amino acids can modulate *C. elegans* lifespan [41]. Therefore, the widespread effect of prohibitin depletion on amino acid metabolism deserves further investigation. Here, we will discuss those that seem more promising. Prohibitin depletion in otherwise wild type animals decreases the content of two branched-chain amino acids, leucine and valine, to the levels encountered in *daf-2(e1370)* mutants, which were not altered upon prohibitin depletion (Fig. 6). The content of these two amino acids in *daf-2(e1370)* mutants is a matter of controversy since previously published data report both higher and lower content of leucine and valine in *daf-2* mutants when compared to wild type worms [33,42,43]. A possible explanation for these discrepancies could be differences in the experimental designs (e.g. stage and bacterial food source) [33,42–44]. Nevertheless, in *daf-2* mutants the changes in the content of these amino acids were found to be entirely DAF-16 dependent [43]. Similar to the effect on the pool of branched amino acids, prohibitin depletion leads to a more pronounced reduction in the content of glutamate and aspartate, in otherwise wild type worms than in *daf-2(e1370)* mutants. Glutamate, through its role in aminotransferase reactions, is at the crossroads of amino acid metabolism, making it of fundamental importance in the synthesis and degradation of other amino acids. Besides, glutamate metabolism is important in multiple other metabolic pathways, and in particular in the replenishing of the tricarboxylic acid (TCA) cycle through its oxidative deamination in mitochondria by glutamate dehydrogenase to α-ketoglutarate (reviewed in [45]). Strikingly, α-ketoglutarate was recently found to extend the lifespan of adult wild type worms in a concentration-dependent manner through the modulation of ATP synthase activity and independently of DAF-16 [46]. Similar to glutamate, aspartate balances the content of the TCA cycle metabolite malate. Previously, the levels of both glutamate and aspartate were shown to strongly decrease in mitochondrial respiratory chain mutants [47]. Consistent with the idea that these changes are, at least partially, related to a perturbation at level of the TCA cycle, we found that the levels of succinate follows a similar trend than those two amino acids. Curiously, it has been reported that *daf-2* mutants shift metabolism away from the TCA cycle towards the glyoxylate cycle [22,43,48], a variation of the TCA cycle that bypasses the decarboxylation steps, and which among other things, enables the interconversion of fats and carbohydrates [49]. This process may be required for the transport of energy between different tissues and has been suggested to be done, among other ways, through trehalose [50]. We identified the disaccharide trehalose as one metabolite very important in the distinction of the different metabolic profiles. Trehalose is an important storage molecule that has been suggested to be a longevity assurance sugar in *C. elegans* [51], and is considered to be a stress responsive metabolite. Trehalose has been proposed to play a role against oxidative and osmotic stresses and in protein stabilization [52–54]. As previously described [33,43], trehalose accumulates to higher levels in *daf-2* mutants than in wild type animals in a DAF-16 independent manner [43]. Prohibitin depletion induces an accumulation of trehalose in both wild type worms and *daf-2(e1370)* mutants, although the levels achieved in wild type worms upon prohibitin depletion do not reach the trehalose levels of *daf-2* mutants in the presence of prohibitin.

Besides the shift towards the glyoxylate shunt, it has been reported that *daf-2* mutants undergo a major metabolic reorganization leading to higher metabolic efficiency [51,55]. Moreover, it has been suggested that a metabolic reorganization at the level of fermentative metabolism, such as lactate and pyruvate fermentation, may contribute to promote longevity [56]. Accordingly, we find significant differences upon prohibitin depletion between wild type worms and *daf-2(e1370)* mutants in the levels of both lactate and the pyruvate-related amino acid alanine. In particular, although the changes in either of the genetic backgrounds are small and not statistically significant, prohibitin depletion has an opposing effect in the content of those two metabolites (Fig. 6B) leading to statistically significant differences between the two genetic backgrounds. Strikingly, an inverse correlation between alanine levels and yeast chorological lifespan has been reported [34]. Moreover, it was shown that the enzyme that metabolises alanine is critical for maintaining normal yeast lifespan [34]. Overall, the opposing effect in the level of both lactate and the pyruvate-related amino acid alanine suggests a difference upon prohibitin depletion in the management of fermentative metabolism in the two genetic backgrounds. Further studies are needed to clarify the occurrence of an alteration in fermentative metabolism in *daf-2* mutants [57] and the impact of prohibitin on it.

In light of the above observations, how can we understand the opposing effect of prohibitin on the longevity of wild type and *daf-2(e1370)* mutants? Given the intertwined regulation of reproduction, fat metabolism and ageing [58], one possibility is that the longevity phenotypes elicited by prohibitin depletion are mediated by a differentially altered germline in both genetic backgrounds. However, several evidences strongly suggest otherwise. First, prohibitin depletion leads to sterility in both wild type and insulin signalling mutants, yet conferring opposite longevity phenotypes [6]. Second, the nuclear hormone receptor NHR-49 is essential for the lifespan extension of germline-ablated animals [59]. Nevertheless, prohibitin depletion extends the lifespan of *nhr-49* mutants [6]. Third, the nuclear hormone receptor DAF-12 is also required for the lifespan extension of germline ablated animals [60] and prohibitin depletion extends the lifespan of *daf-2(e1370)*;*daf-12(m20)* double mutants (our unpublished data). These observations strongly indicate that the effect of prohibitin on longevity is independent of the germline. The more pronounced effect of prohibitin depletion on the metabolic profiles of wild type animals as compared to *daf-2(e1370)* mutants suggests that the metabolic reorganization occurred in *daf-2* [22,23] makes these animals more robust to the changes induced by prohibitin depletion. Therefore, we favour the scenario where, in the context of a *daf-2* mutant reorganized metabolic network, the changes induced by prohibitin depletion have a beneficial and synergistic effect on the worm longevity, while having a detrimental effect on the longevity of wild type animals (Fig. 6C). Prohibitin deficiency has an impact on the fatty acid composition, amino acid and carbohydrate metabolism on both wild type and *daf-2(e1370)* genetic backgrounds. Moreover, we observe changes that are consistent with a differential effect at the level of fermentative metabolism, which may be partially responsible for the longevity phonotypes (Fig. 6C). *daf-2* mutants show an increase in mitochondrial fermentative malate dismutation, which contributes to their increased lifespan expectancy [47,56,61]. Malate dismutation in *C. elegans* needs the activity of the glyoxylate cycle and fatty-acid derived acetyl-CoA [47,62]. In yeast, impairment of the electron transport chain (ETC) triggers the activity of anaplerotic pathways, like the glyoxylate cycle and fatty acid β-oxidation, to feed intermediates into the TCA cycle [63]. Interestingly, prohibitin deficiency has been linked to electron transfer impairment at complex I [64,65] and several links between ETC complexes and prohibitins have been described [66,67]. It has been proposed that worm mutants with an affected ETC [56], like *clk-1* mutants, activate fermentative metabolism in an inefficient way, while in *daf-2* mutants fermentative metabolism is constitutively active. Therefore, in *clk-1*;*daf-2* double mutants there is a powerful increase in fermentative metabolism, with a concomitant reduction in the generation of ROS leading to a beneficial outcome in terms of longevity [56]. In a similar way, we hypothesized that in prohibitin-depleted *daf-2* mutants there is an increase in fermentative metabolism, while in prohibitin-depleted worms there is not (Fig. 6C). Consistent with this idea, prohibitin depletion increased ROS levels in otherwise wild type worms, while it decreased ROS in *daf-2* mutants [6]. Thus, a plausible explanation for the synergetic effect of prohibitin depletion on the longevity of *daf-2* mutants is the observed additive effect of prohibitin depletion in the fatty acid composition of *daf-2(e1370)* mutants, which in the context of a reorganized metabolic network, namely a more efficient fermentative metabolism, may contribute to the positive outcome in terms of longevity (Fig. 6C). Nevertheless, we do not rule out the possibility of a differential effect on other metabolic pathways and/or specific classes of lipids. The differences in longevity outcome might also be understood in the scope of the observation that prohibitin deficiency has a strong effect on glutamate metabolism, which has been shown to be linked to insulin secretion in β-cells through glutamate dehydrogenase [68–70]. Thus, if this connection also exists in *C. elegans*, the strong effect of prohibitin in the levels of glutamate observed in both genetic backgrounds may give a plausible metabolic connection between prohibitin and the IIS pathway. The same effect of prohibitin in the levels of glutamate may have a complete different outcome in animals with an intact or with a compromised IIS pathway, with implications on the longevity of the worms.

## 5 Conclusion

The effect of prohibitin depletion on the fatty acid and ^1^H NMR metabolic profiles of whole worm extracts revealed broad metabolic changes. RNAi targeting either *phb-1* or *phb-2* had a similar effect on the metabolic profiles of otherwise wild type worms, arguing in favour of PHB-1 and PHB-2 functioning together within the mitochondrial prohibitin complex. Moreover, the alterations induced by prohibitin depletion in the metabolic profiles of otherwise wild type worms, suggests that along time the metabolome changes in a prohibitin-dependent manner. Strikingly, prohibitin depletion affects more deeply fatty acid composition, and amino acid and carbohydrate metabolism in otherwise wild type than in *daf-2* mutant worms. Additionally, RNAi targeting of *phb-1* in *daf-2* mutants had an additive effect in some of the changes observed in the metabolic profile of *daf-2* mutants compared to wild type animals. Altogether, this work adds significant data that provide crucial insights to understand how mitochondria and the insulin/insulin-like signalling pathway interact to determine longevity. Moreover, it stresses the importance of further metabolic studies, involving more global mass spectrometry-based approaches, to obtain a more complete picture of the metabolic changes elicited to prolong life. Herein, we describe the first global molecular characterisation of the effect of prohibitin in the *C. elegans* metabolome, providing extremely valuable molecular insights towards understanding the opposing effect of prohibitin depletion in longevity.

The following are the supplementary data related to this article.

Table S1 Fatty acid composition in wild type (N2) worms upon prohibitin depletion. N2 worms were grown in liquid medium and analysed at A) fourth larval stage of development (L4) and B) young adult (YA) stage. μ corresponds to the average value, while δ to the standard deviation and CV to the coefficient of variation (δ/μ ∗ 100) of the content of the different fatty acids. P-values are derived from t-test analysis.

Table S2 Analysis of the composition of different metabolites present in wild type (N2) worms upon prohibitin depletion. N2 worms were grown in liquid medium and analysed at A) fourth larval stage of development (L4) and B) young adult (YA) stage. μ corresponds to the average value, while δ to the standard deviation and CV to the coefficient of variation (δ/μ ∗ 100) of the metabolite content (characteristic bin). P-values are derived from t-test analysis.

Table S3 Fatty acid composition in wild type (N2) and *daf-2(e1370)* worms upon prohibitin depletion. Worms were grown on plate and analysed at young adult (YA) stage. μ corresponds to the average value, while δ to the standard deviation and CV to the coefficient of variation (δ/μ ∗ 100) of the content of the different fatty acids. P-values are derived from t-test analysis.

Table S4 Analysis of the composition of different metabolites present in wild type (N2) and *daf-2(e1370)* worms upon prohibitin depletion. Worms were grown on plate and analysed at young adult (YA) stage. μ corresponds to the average value, while δ to the standard deviation and CV to the coefficient of variation (δ/μ ∗ 100) of the metabolite content (characteristic bin). P-values are derived from t-test analysis.

## Conflict of interest

The authors declare that they have no conflict of interest.
