# Supplementary material for: Analysis of the effect of the mitochondrial prohibitin complex, a context-dependent modulator of longevity, on the C. elegans metabolome
Source: Biochim Biophys Acta. 2015 Nov;1847(11):1457–68. doi: 10.1016/j.bbabio.2015.06.003 (PMC4580209; doi:10.1016/j.bbabio.2015.06.003)
Supplement: Table S1 — Fatty acid composition in wild type (N2) worms upon prohibitin depletion. N2 worms were grown in liquid medium and analysed at A) fourth larval stage of development (L4) and B) young adult (YA) stage. μ corresponds to the average value, while δ to the standard deviation and CV to the coefficient of variation (δ/μ ∗ 100) of the content of the different fatty acids. P-values are derived from t-test analysis. [file mmc1.docx]

**Table S1A**

|  | **Control RNAi** | | | ***phb-1(RNAi)*** | | | ***phb-2(RNAi)*** | | | **P-value** | |
| --- | --- | --- | --- | --- | --- | --- | --- | --- | --- | --- | --- |
| **Fatty Acids** | **µ** | **δ** | **CV (%)** | **µ** | **δ** | **CV (%)** | **µ** | **δ** | **CV (%)** | **Control RNAi**  **vs**  ***phb-1(RNAi)*** | **Control RNAi**  **vs**  ***phb-2(RNAi)*** |
| **C14:0** | 0.8875 | 0.0742 | 8.3557 | 1.4538 | 0.3085 | 21.2192 | 1.4525 | 0.2177 | 14.9878 | **0.0195** | **0.0046** |
| **C14:1** | 2.1708 | 0.1385 | 6.3788 | 3.2976 | 0.8114 | 24.6063 | 3.0151 | 0.2999 | 9.9458 | **0.0489** | **0.0026** |
| **C16:0** | 7.2697 | 0.3806 | 5.2356 | 7.2684 | 0.3343 | 4.5994 | 7.7372 | 0.9841 | 12.7192 | 0.9960 | 0.4149 |
| **C16:1** | 7.3115 | 1.4456 | 19.7710 | 6.9228 | 2.5909 | 37.4260 | 7.6762 | 3.2803 | 42.7336 | 0.8017 | 0.8461 |
| **C18:0** | 9.0919 | 0.9097 | 10.0053 | 9.7007 | 0.8189 | 8.4416 | 9.8882 | 1.3791 | 13.9471 | 0.3492 | 0.3675 |
| **C18:1n9c** | 4.9060 | 0.6894 | 14.0527 | 8.5580 | 0.8361 | 9.7698 | 7.8005 | 2.1696 | 27.8136 | **0.0002** | 0.0537 |
| **C18:1n7** | 23.6552 | 4.1059 | 17.3574 | 23.2453 | 3.2290 | 13.8909 | 19.9083 | 8.3202 | 41.7928 | 0.8794 | 0.4509 |
| **C18:2n6c** | 2.8212 | 0.8415 | 29.8289 | 3.6201 | 1.0673 | 29.4830 | 5.0377 | 3.5873 | 71.2102 | 0.2754 | 0.2892 |
| **C20:0** | 2.1394 | 0.2083 | 9.7386 | 2.3828 | 0.1582 | 6.6394 | 2.5257 | 0.4922 | 19.4881 | 0.1025 | 0.2038 |
| **C20:3n6** | 9.7306 | 3.2925 | 33.8365 | 7.6256 | 1.6692 | 21.8898 | 8.3565 | 1.4632 | 17.5093 | 0.2981 | 0.4769 |
| **C20:4n6** | 2.3852 | 0.4450 | 18.6560 | 2.8399 | 0.3301 | 11.6242 | 3.1647 | 0.3599 | 11.3720 | 0.1426 | **0.0271** |
| **C20:4n3** | 8.6531 | 0.7545 | 8.7197 | 7.5919 | 1.0243 | 13.4920 | 7.2120 | 1.9336 | 26.8114 | 0.1371 | 0.2216 |
| **C20:5n3** | 18.9778 | 1.2636 | 6.6581 | 15.4931 | 1.5525 | 10.0206 | 16.2255 | 1.5856 | 9.7723 | **0.0088** | **0.0277** |

**Table S1B**

|  | **Control RNAi** | | | ***phb-1(RNAi)*** | | | ***phb-2(RNAi)*** | | | **P-value** | |
| --- | --- | --- | --- | --- | --- | --- | --- | --- | --- | --- | --- |
| **Fatty Acids** | **µ** | **δ** | **CV (%)** | **µ** | **δ** | **CV (%)** | **µ** | **δ** | **CV (%)** | **Control RNAi**  **vs**  ***phb-1(RNAi)*** | **Control RNAi**  **vs**  ***phb-2(RNAi)*** |
| **C14:0** | 0.5948 | 0.2323 | 39.0462 | 2.0733 | 0.3912 | 18.8704 | 2.7587 | 0.8622 | 31.2539 | **0.0005** | **0.0059** |
| **C14:1** | 2.4018 | 0.6341 | 26.3999 | 4.3644 | 0.8329 | 19.0846 | 4.6243 | 0.9887 | 21.3801 | **0.0064** | **0.0072** |
| **C16:0** | 6.1777 | 0.7624 | 12.3417 | 7.7181 | 0.7246 | 9.3887 | 9.6732 | 1.0377 | 10.7277 | **0.0191** | **0.0008** |
| **C16:1** | 4.2671 | 1.6069 | 37.6588 | 10.3190 | 2.7586 | 26.7335 | 9.8301 | 3.5459 | 36.0720 | **0.0079** | **0.0313** |
| **C18:0** | 8.7671 | 0.6965 | 7.9448 | 8.9899 | 1.0633 | 11.8275 | 10.1763 | 1.3839 | 13.5989 | 0.7364 | 0.1196 |
| **C18:1n9c** | 5.3219 | 0.6842 | 12.8565 | 8.8068 | 0.2611 | 2.9647 | 9.8187 | 1.3246 | 13.4907 | **0.0002** | **0.0009** |
| **C18:1n7** | 23.0408 | 4.5948 | 19.9419 | 24.5853 | 2.1874 | 8.8972 | 21.9036 | 1.8293 | 8.3514 | 0.5671 | 0.6641 |
| **C18:2n6c** | 6.5663 | 0.5014 | 7.6364 | 3.0078 | 1.0746 | 35.7283 | 3.4267 | 1.3807 | 40.2922 | **0.0012** | **0.0078** |
| **C20:0** | 1.8773 | 0.2997 | 15.9669 | 1.5770 | 0.1553 | 9.8466 | 1.8287 | 0.4384 | 23.9730 | 0.1255 | 0.8598 |
| **C20:3n6** | 7.9835 | 1.3765 | 17.2417 | 5.8624 | 0.4140 | 7.0617 | 5.2996 | 0.4110 | 7.7555 | **0.0342** | **0.0150** |
| **C20:4n6** | 2.0948 | 0.5442 | 25.9798 | 2.6846 | 0.1722 | 6.4158 | 2.3532 | 0.3877 | 16.4769 | 0.0961 | 0.4637 |
| **C20:4n3** | 10.0138 | 0.6200 | 6.1919 | 6.0139 | 0.7809 | 12.9851 | 5.2255 | 1.4601 | 27.9412 | **0.0001** | **0.0014** |
| **C20:5n3** | 20.8931 | 1.2139 | 5.8103 | 13.9975 | 1.4797 | 10.5715 | 13.0814 | 0.9954 | 7.6090 | **0.0001** | **0.0000** |
