# Supplementary material for: Analysis of the effect of the mitochondrial prohibitin complex, a context-dependent modulator of longevity, on the C. elegans metabolome
Source: Biochim Biophys Acta. 2015 Nov;1847(11):1457–68. doi: 10.1016/j.bbabio.2015.06.003 (PMC4580209; doi:10.1016/j.bbabio.2015.06.003)
Supplement: Table S2 — Analysis of the composition of different metabolites present in wild type (N2) worms upon prohibitin depletion. N2 worms were grown in liquid medium and analysed at A) fourth larval stage of development (L4) and B) young adult (YA) stage. μ corresponds to the average value, while δ to the standard deviation and CV to the coefficient of variation (δ/μ ∗ 100) of the metabolite content (characteristic bin). P-values are derived from t-test analysis. [file mmc2.docx]

Table S2 A

|  | **Control RNAi** | | | ***phb-1(RNAi)*** | | | ***phb-2(RNAi)*** | | | **P-value** | |
| --- | --- | --- | --- | --- | --- | --- | --- | --- | --- | --- | --- |
| **Metabolite** | **µ** | **ᵟ** | **CV (%)** | **µ** | **ᵟ** | **CV (%)** | **µ** | **ᵟ** | **CV (%)** | **Control RNAi**  **vs**  ***phb-1(RNAi)*** | **Control RNAi**  **vs**  ***phb-2(RNAi)*** |
| **Leucine** | 0.0061 | 0.0003 | 4.3473 | 0.0042 | 0.0006 | 13.4614 | 0.0048 | 0.0010 | 21.8575 | **0.0011** | 0.0637 |
| **Valine** | 0.0014 | 0.0002 | 11.9356 | 0.0010 | 0.0001 | 8.0163 | 0.0017 | 0.0010 | 57.6581 | **0.0078** | 0.5973 |
| **Lactate** | 0.0005 | 0.0001 | 18.5532 | 0.0004 | 0.0001 | 26.7582 | 0.0004 | 0.0001 | 18.4217 | 0.6074 | 0.1027 |
| **Alanine** | 0.0229 | 0.0028 | 12.2665 | 0.0352 | 0.0059 | 16.7539 | 0.0313 | 0.0030 | 9.6791 | **0.0102** | **0.0036** |
| **Glutamate** | 0.0040 | 0.0004 | 9.4271 | 0.0027 | 0.0005 | 17.1484 | 0.0034 | 0.0008 | 22.9009 | **0.0025** | 0.1736 |
| **Glutamine** | 0.0022 | 0.0001 | 5.2873 | 0.0022 | 0.0002 | 10.4215 | 0.0022 | 0.0002 | 10.4215 | 0.6780 | 0.6780 |
| **Succinate** | 0.0028 | 0.0006 | 19.9768 | 0.0029 | 0.0014 | 46.9438 | 0.0021 | 0.0005 | 21.9865 | 0.8947 | 0.0876 |
| **Beta-alanine** | 0.0018 | 0.0001 | 5.0341 | 0.0017 | 0.0001 | 6.4208 | 0.0016 | 0.0002 | 12.1321 | 0.2831 | 0.0989 |
| **Cystathionine** | 0.0019 | 0.0002 | 8.9946 | 0.0030 | 0.0003 | 9.8980 | 0.0029 | 0.0002 | 7.7876 | **0.0006** | **0.0002** |
| **Aspartate** | 0.0002 | 0.0000 | 9.7877 | 0.0002 | 0.0000 | 19.7493 | 0.0002 | 0.0000 | 13.6246 | 0.8374 | 0.4136 |
| **Asparagine** | 0.0003 | 0.0000 | 12.9352 | 0.0005 | 0.0001 | 26.7332 | 0.0005 | 0.0001 | 26.1807 | **0.0327** | **0.0328** |
| **Lysine** | 0.0016 | 0.0001 | 4.3747 | 0.0019 | 0.0002 | 10.0631 | 0.0017 | 0.0002 | 12.7457 | 0.0533 | 0.4045 |
| **Ornithine** | 0.0007 | 0.0000 | 6.6236 | 0.0010 | 0.0001 | 13.7883 | 0.0010 | 0.0001 | 8.6750 | **0.0108** | **0.0023** |
| **Arginine** | 0.0014 | 0.0001 | 9.4995 | 0.0019 | 0.0002 | 8.0571 | 0.0018 | 0.0003 | 14.7148 | **0.0007** | **0.0263** |
| **Betaine** | 0.0080 | 0.0009 | 11.9023 | 0.0083 | 0.0016 | 19.6181 | 0.0098 | 0.0012 | 12.0631 | 0.6980 | **0.0427** |
| **Glycine** | 0.0043 | 0.0004 | 8.9126 | 0.0045 | 0.0011 | 23.7902 | 0.0043 | 0.0007 | 15.2975 | 0.7061 | 0.9888 |
| **Glycerol** | 0.0140 | 0.0014 | 9.6737 | 0.0110 | 0.0014 | 12.4038 | 0.0124 | 0.0023 | 18.8265 | **0.0142** | 0.2673 |
| **Threonine** | 0.0010 | 0.0001 | 8.8536 | 0.0009 | 0.0001 | 11.0414 | 0.0009 | 0.0001 | 13.3335 | **0.0429** | 0.1578 |
| **Glutathione** | 0.0003 | 0.0001 | 28.5748 | 0.0003 | 0.0001 | 33.9795 | 0.0002 | 0.0001 | 32.2124 | 0.2714 | 0.6140 |
| **Glucose** | 0.0002 | 0.0000 | 13.5436 | 0.0003 | 0.0001 | 34.4213 | 0.0003 | 0.0001 | 23.4334 | 0.1490 | **0.0657** |
| **Trehalose** | 0.0009 | 0.0002 | 24.0926 | 0.0030 | 0.0007 | 23.8805 | 0.0027 | 0.0004 | 15.9664 | **0.0030** | **0.0003** |
| **Allantoin** | 0.0001 | 0.0000 | 16.9019 | 0.0002 | 0.0000 | 22.8436 | 0.0002 | 0.0001 | 36.5752 | **0.0250** | 0.1010 |
| **NAD^+^** | 0.0002 | 0.0000 | 1.0798 | 0.0002 | 0.0000 | 12.8067 | 0.0002 | 0.0000 | 13.3289 | 0.1545 | 0.2623 |
| **Fumarate** | 0.0000 | 0.0000 | 25.7217 | 0.0000 | 0.0000 | 19.4722 | 0.0000 | 0.0000 | 45.6844 | 0.5736 | 0.8125 |
| **Tyrosine** | 0.0002 | 0.0000 | 9.6824 | 0.0004 | 0.0001 | 19.0183 | 0.0003 | 0.0000 | 9.9083 | **0.0203** | **0.0043** |
| **Histidine** | 0.0007 | 0.0000 | 4.0244 | 0.0007 | 0.0000 | 3.5717 | 0.0006 | 0.0001 | 14.4917 | 0.4515 | 0.1931 |
| **Phenylalanine** | 0.0004 | 0.0000 | 6.3600 | 0.0004 | 0.0001 | 15.2014 | 0.0004 | 0.0001 | 14.5708 | 0.3651 | 0.2642 |
| **Tryptophan** | 0.0001 | 0.0000 | 9.2403 | 0.0002 | 0.0000 | 13.3709 | 0.0002 | 0.0000 | 8.7117 | **0.0067** | **0.0027** |
| **AMP** | 0.0004 | 0.0001 | 32.7886 | 0.0004 | 0.0001 | 13.3429 | 0.0005 | 0.0001 | 23.2005 | 0.4821 | 0.2083 |

Table S2 B

|  | **Control RNAi** | | | ***phb-1(RNAi)*** | | | ***phb-2(RNAi)*** | | | **P-value** | |
| --- | --- | --- | --- | --- | --- | --- | --- | --- | --- | --- | --- |
| **Metabolite** | **µ** | **ᵟ** | **CV (%)** | **µ** | **ᵟ** | **CV (%)** | **µ** | **ᵟ** | **CV (%)** | **Control RNAi**  **vs**  ***phb-1(RNAi)*** | **Control RNAi**  **vs**  ***phb-2(RNAi)*** |
| **Leucine** | 0.0062 | 0.0001 | 2.2983 | 0.0043 | 0.0002 | 4.5678 | 0.0048 | 0.0003 | 6.0056 | **0.0000** | **0.0001** |
| **Valine** | 0.0038 | 0.0008 | 21.9798 | 0.0012 | 0.0002 | 12.0520 | 0.0016 | 0.0004 | 24.7768 | **0.0031** | **0.0037** |
| **Lactate** | 0.0005 | 0.0001 | 12.6231 | 0.0005 | 0.0001 | 19.3979 | 0.0004 | 0.0000 | 6.4197 | 0.9408 | 0.2697 |
| **Alanine** | 0.0251 | 0.0016 | 6.5414 | 0.0306 | 0.0025 | 8.2619 | 0.0342 | 0.0025 | 7.3562 | **0.0088** | **0.0006** |
| **Glutamate** | 0.0047 | 0.0003 | 6.8147 | 0.0030 | 0.0003 | 10.1548 | 0.0035 | 0.0003 | 9.9039 | **0.0001** | **0.0009** |
| **Glutamine** | 0.0024 | 0.0002 | 6.3429 | 0.0031 | 0.0004 | 11.8180 | 0.0027 | 0.0005 | 18.7868 | **0.0133** | 0.2782 |
| **Succinate** | 0.0038 | 0.0010 | 26.6781 | 0.0028 | 0.0006 | 20.4027 | 0.0023 | 0.0004 | 17.9623 | 0.1324 | **0.0388** |
| **Beta-alanine** | 0.0019 | 0.0002 | 11.8984 | 0.0012 | 0.0001 | 6.8452 | 0.0013 | 0.0002 | 14.4191 | **0.0020** | **0.0024** |
| **Cystathionine** | 0.0024 | 0.0003 | 10.8149 | 0.0023 | 0.0002 | 10.8592 | 0.0024 | 0.0001 | 6.0055 | 0.4470 | 0.9606 |
| **Aspartate** | 0.0001 | 0.0000 | 18.5829 | 0.0002 | 0.0000 | 12.0415 | 0.0002 | 0.0000 | 13.4362 | 0.0638 | 0.1831 |
| **Asparagine** | 0.0002 | 0.0000 | 11.9577 | 0.0004 | 0.0001 | 12.7900 | 0.0004 | 0.0001 | 25.3624 | **0.0006** | **0.0165** |
| **Lysine** | 0.0014 | 0.0001 | 7.2077 | 0.0014 | 0.0001 | 5.2750 | 0.0015 | 0.0003 | 19.0171 | 0.3613 | 0.5831 |
| **Ornithine** | 0.0009 | 0.0002 | 22.6506 | 0.0010 | 0.0002 | 20.4914 | 0.0011 | 0.0002 | 19.0404 | 0.4087 | 0.2279 |
| **Arginine** | 0.0017 | 0.0001 | 7.6919 | 0.0021 | 0.0001 | 5.0731 | 0.0020 | 0.0001 | 6.1859 | **0.0022** | **0.0060** |
| **Betaine** | 0.0095 | 0.0008 | 8.7863 | 0.0094 | 0.0029 | 30.3884 | 0.0103 | 0.0016 | 15.6324 | 0.9505 | 0.4083 |
| **Glycine** | 0.0061 | 0.0008 | 13.1247 | 0.0058 | 0.0014 | 23.8205 | 0.0050 | 0.0004 | 8.1331 | 0.7500 | **0.0463** |
| **Glycerol** | 0.0210 | 0.0018 | 8.4156 | 0.0154 | 0.0015 | 9.4672 | 0.0153 | 0.0020 | 13.3907 | **0.0013** | **0.0029** |
| **Threonine** | 0.0013 | 0.0001 | 4.3523 | 0.0007 | 0.0001 | 7.6394 | 0.0009 | 0.0001 | 15.6063 | **0.0000** | **0.0020** |
| **Glutathione** | 0.0002 | 0.0001 | 36.0944 | 0.0002 | 0.0001 | 34.3227 | 0.0003 | 0.0000 | 11.9316 | 0.6777 | 0.4323 |
| **Glucose** | 0.0006 | 0.0001 | 18.4678 | 0.0005 | 0.0001 | 16.1050 | 0.0005 | 0.0001 | 11.5258 | 0.2177 | 0.1299 |
| **Trehalose** | 0.0018 | 0.0003 | 13.6942 | 0.0037 | 0.0002 | 4.4344 | 0.0040 | 0.0009 | 22.1962 | **0.0000** | **0.0064** |
| **Allantoin** | 0.0003 | 0.0001 | 27.0058 | 0.0002 | 0.0000 | 21.0642 | 0.0002 | 0.0000 | 19.2804 | 0.1009 | **0.0171** |
| **NAD^+^** | 0.0002 | 0.0000 | 9.6286 | 0.0001 | 0.0000 | 9.0158 | 0.0002 | 0.0000 | 5.6950 | 0.2543 | 0.3562 |
| **Fumarate** | 0.0000 | 0.0000 | 31.0966 | 0.0000 | 0.0000 | 23.4602 | 0.0000 | 0.0000 | 21.7217 | 0.0896 | 0.2081 |
| **Tyrosine** | 0.0003 | 0.0000 | 9.3449 | 0.0003 | 0.0000 | 14.2197 | 0.0003 | 0.0000 | 12.9262 | 0.1144 | 0.2799 |
| **Histidine** | 0.0006 | 0.0000 | 5.5878 | 0.0007 | 0.0000 | 5.3811 | 0.0006 | 0.0001 | 9.3213 | 0.1490 | 0.6513 |
| **Phenylalanine** | 0.0005 | 0.0001 | 15.1968 | 0.0003 | 0.0000 | 15.5093 | 0.0003 | 0.0000 | 15.5093 | **0.0013** | **0.0013** |
| **Tryptophan** | 0.0001 | 0.0000 | 15.3149 | 0.0001 | 0.0000 | 11.2416 | 0.0002 | 0.0000 | 26.0778 | 0.5753 | 0.2118 |
| **AMP** | 0.0006 | 0.0001 | 9.9343 | 0.0004 | 0.0001 | 19.3815 | 0.0005 | 0.0001 | 15.8588 | **0.0090** | **0.0220** |
