# Supplementary material for: Analysis of the effect of the mitochondrial prohibitin complex, a context-dependent modulator of longevity, on the C. elegans metabolome
Source: Biochim Biophys Acta. 2015 Nov;1847(11):1457–68. doi: 10.1016/j.bbabio.2015.06.003 (PMC4580209; doi:10.1016/j.bbabio.2015.06.003)
Supplement: Table S3 — Fatty acid composition in wild type (N2) and daf-2(e1370) worms upon prohibitin depletion. Worms were grown on plate and analysed at young adult (YA) stage. μ corresponds to the average value, while δ to the standard deviation and CV to the coefficient of variation (δ/μ ∗ 100) of the content of the different fatty acids. P-values are derived from t-test analysis. [file mmc3.docx]

**Table S3**

|  |  |  |  |  |  |  |  |  |  |  |  |  | **P-value** | | | |
| --- | --- | --- | --- | --- | --- | --- | --- | --- | --- | --- | --- | --- | --- | --- | --- | --- |
|  | **Control RNAi** | | | ***phb-1(RNAi)*** | | | ***daf-2(e1370)*; Control RNAi** | | | ***daf-2(e1370)*; *phb-1(RNAi)*** | | | **N2** | ***daf-2(e1370)*** | **Control RNAi** | ***phb-1(RNAi)*** |
| **Fatty acids** | **µ** | **ᵟ** | **CV (%)** | **µ** | **ᵟ** | **CV (%)** | **µ** | **ᵟ** | **CV (%)** | **µ** | **ᵟ** | **CV (%)** | **Control RNAi**  **vs**  ***phb-1(RNAi)*** | **Control RNAi**  **vs**  ***phb-1(RNAi)*** | **N2**  **vs**  ***daf-2(e1370)*** | **N2**  **vs**  ***daf-2(e1370)*** |
| **C14:0** | 0.5597 | 0.1387 | 24.7840 | 1.1355 | 0.5247 | 46.2077 | 2.3779 | 0.2258 | 9.4971 | 2.8708 | 0.5662 | 19.7243 | 0.1521 | 0.2352 | **0.0001** | **0.0081** |
| **C14:1** | 1.9426 | 0.2618 | 13.4747 | 2.6340 | 0.7522 | 28.5563 | 3.5000 | 0.5104 | 14.5830 | 3.6276 | 0.6879 | 18.9634 | 0.2124 | 0.8057 | **0.0070** | 0.1427 |
| **C16:0** | 6.0308 | 0.9154 | 15.1793 | 5.3324 | 0.7832 | 14.6880 | 7.6921 | 1.1174 | 14.5264 | 7.3267 | 0.7293 | 9.9542 | 0.3549 | 0.6547 | 0.0953 | **0.0181** |
| **C16:1** | 5.6535 | 0.3908 | 6.9117 | 6.6854 | 0.8554 | 12.7944 | 11.5916 | 0.7170 | 6.1857 | 13.8811 | 0.6387 | 4.6009 | 0.1267 | **0.0063** | **0.0001** | **0.0000** |
| **C18:0** | 8.3117 | 0.1664 | 2.0024 | 6.9627 | 0.8798 | 12.6364 | 6.4541 | 0.6213 | 9.6265 | 5.5756 | 0.5762 | 10.3351 | 0.0743 | 0.1230 | **0.0111** | 0.0694 |
| **C18:1n9c** | 5.9213 | 1.3012 | 21.9756 | 7.0731 | 0.3698 | 5.2284 | 6.6377 | 0.6140 | 9.2509 | 7.5473 | 0.7996 | 10.5942 | 0.2245 | 0.1724 | 0.4342 | 0.4013 |
| **C18:1n7** | 29.6938 | 0.5944 | 2.0016 | 31.6922 | 2.1492 | 6.7814 | 30.9527 | 1.7586 | 5.6815 | 33.7609 | 1.2237 | 3.6246 | 0.2065 | 0.0689 | 0.3105 | 0.2099 |
| **C18:2n6c** | 4.2996 | 0.2526 | 5.8738 | 7.2747 | 0.5178 | 7.1173 | 5.8875 | 0.4677 | 7.9439 | 6.9481 | 0.1940 | 2.7916 | **0.0006** | **0.0222** | **0.0045** | 0.3664 |
| **C20:0** | 2.2191 | 0.1619 | 7.2945 | 1.6237 | 0.0430 | 2.6477 | 1.4682 | 0.0384 | 2.6132 | 1.1042 | 0.0234 | 2.1186 | **0.0058** | **0.0000** | **0.0030** | **0.0000** |
| **C20:3n6** | 6.2501 | 0.2778 | 4.4455 | 5.7452 | 0.0938 | 1.6325 | 4.7645 | 0.1788 | 3.7537 | 3.7552 | 0.1289 | 3.4338 | **0.0453** | **0.0003** | **0.0005** | **0.0000** |
| **C20:4n6** | 2.3982 | 0.2014 | 8.3993 | 2.9399 | 0.2039 | 6.9361 | 1.4382 | 0.0968 | 6.7301 | 1.4179 | 0.1250 | 8.8191 | **0.0170** | 0.8324 | **0.0013** | **0.0001** |
| **C20:4n3** | 7.3086 | 0.3159 | 4.3218 | 5.5802 | 0.1715 | 3.0732 | 5.4433 | 0.2327 | 4.2752 | 3.8185 | 0.2617 | 6.8525 | **0.0006** | **0.0002** | **0.0003** | **0.0002** |
| **C20:5n3** | 19.4111 | 0.4766 | 2.4553 | 15.3211 | 0.6529 | 4.2613 | 11.7923 | 0.4127 | 3.4994 | 8.3662 | 0.4126 | 4.9317 | **0.0002** | **0.0001** | **0.0000** | **0.0000** |
