# Supplementary material for: Analysis of the effect of the mitochondrial prohibitin complex, a context-dependent modulator of longevity, on the C. elegans metabolome
Source: Biochim Biophys Acta. 2015 Nov;1847(11):1457–68. doi: 10.1016/j.bbabio.2015.06.003 (PMC4580209; doi:10.1016/j.bbabio.2015.06.003)
Supplement: Table S4 — Analysis of the composition of different metabolites present in wild type (N2) and daf-2(e1370) worms upon prohibitin depletion. Worms were grown on plate and analysed at young adult (YA) stage. μ corresponds to the average value, while δ to the standard deviation and CV to the coefficient of variation (δ/μ ∗ 100) of the metabolite content (characteristic bin). P-values are derived from t-test analysis. [file mmc4.docx]

Table S4

|  |  |  |  |  |  |  |  |  |  |  |  |  | **P-value** | | | |
| --- | --- | --- | --- | --- | --- | --- | --- | --- | --- | --- | --- | --- | --- | --- | --- | --- |
|  | **Control RNAi** | | | ***phb-1(RNAi)*** | | | ***daf-2(e1370)*; Control RNAi** | | | ***daf-2(e1370)*; *phb-1(RNAi)*** | | | **N2** | ***daf-2(e1370)*** | **Control RNAi** | ***phb-1(RNAi)*** |
| **Metabolite** | **µ** | **ᵟ** | **CV (%)** | **µ** | **ᵟ** | **CV (%)** | **µ** | **ᵟ** | **CV (%)** | **µ** | **ᵟ** | **CV (%)** | **Control RNAi**  **vs**  ***phb-1(RNAi)*** | **control RNAi**  **vs**  ***phb-1(RNAi)*** | **N2**  **vs**  ***daf-2(e1370)*** | **N2**  **vs**  ***daf-2(e1370)*** |
| **Leucine** | 0.0057 | 0.0002 | 3.7230 | 0.0046 | 0.0003 | 6.2050 | 0.0042 | 0.0003 | 6.7422 | 0.0045 | 0.0003 | 6.9274 | **0.0027** | 0.2065 | **0.0004** | 0.6781 |
| **Valine** | 0.0019 | 0.0001 | 6.1175 | 0.0013 | 0.0000 | 3.5170 | 0.0013 | 0.0001 | 5.1588 | 0.0015 | 0.0001 | 6.0010 | **0.0012** | 0.0666 | **0.0008** | **0.0396** |
| **Lactate** | 0.0022 | 0.0002 | 9.4691 | 0.0024 | 0.0001 | 4.8780 | 0.0016 | 0.0001 | 4.9703 | 0.0015 | 0.0003 | 18.6507 | 0.1833 | 0.6248 | **0.0110** | **0.0071** |
| **Alanine** | 0.0266 | 0.0012 | 4.4200 | 0.0278 | 0.0019 | 6.7475 | 0.0241 | 0.0028 | 11.7676 | 0.0220 | 0.0018 | 8.1718 | 0.3995 | 0.3325 | 0.2269 | **0.0085** |
| **Glutamate** | 0.0051 | 0.0004 | 6.8211 | 0.0024 | 0.0003 | 10.8446 | 0.0034 | 0.0003 | 8.1839 | 0.0021 | 0.0001 | 5.8033 | **0.0001** | **0.0014** | **0.0007** | 0.1158 |
| **Glutamine** | 0.0028 | 0.0002 | 6.1463 | 0.0035 | 0.0003 | 8.7028 | 0.0024 | 0.0001 | 2.4970 | 0.0028 | 0.0002 | 7.0042 | **0.0166** | **0.0401** | **0.0209** | **0.0137** |
| **Succinate** | 0.0040 | 0.0003 | 7.5468 | 0.0019 | 0.0004 | 21.6329 | 0.0032 | 0.0003 | 9.0164 | 0.0017 | 0.0009 | 54.4174 | **0.0005** | 0.0544 | **0.0227** | 0.7524 |
| **Beta-alanine** | 0.0012 | 0.0001 | 10.8476 | 0.0007 | 0.0000 | 5.9669 | 0.0009 | 0.0001 | 12.1743 | 0.0006 | 0.0001 | 17.9998 | **0.0039** | **0.0183** | **0.0221** | 0.3795 |
| **Cystathionine** | 0.0007 | 0.0002 | 24.2677 | 0.0005 | 0.0000 | 9.0991 | 0.0011 | 0.0002 | 20.7279 | 0.0012 | 0.0001 | 10.0432 | 0.1526 | 0.4435 | 0.0719 | **0.0009** |
| **Aspartate** | 0.0007 | 0.0001 | 11.2338 | 0.0004 | 0.0001 | 16.0585 | 0.0004 | 0.0001 | 16.0585 | 0.0003 | 0.0000 | 7.5368 | **0.0066** | 0.0739 | **0.0066** | 0.0739 |
| **Asparagine** | 0.0003 | 0.0000 | 2.8261 | 0.0004 | 0.0001 | 17.4159 | 0.0004 | 0.0001 | 18.8426 | 0.0006 | 0.0001 | 15.6265 | **0.0483** | 0.0548 | 0.0768 | 0.0809 |
| **Lysine** | 0.0025 | 0.0002 | 9.3464 | 0.0024 | 0.0000 | 1.7605 | 0.0020 | 0.0001 | 3.4559 | 0.0019 | 0.0001 | 6.5698 | 0.5260 | 0.2504 | **0.0362** | **0.0047** |
| **Ornithine** | 0.0011 | 0.0000 | 4.0855 | 0.0010 | 0.0000 | 1.5962 | 0.0010 | 0.0000 | 4.2810 | 0.0015 | 0.0003 | 21.5085 | **0.0067** | 0.0674 | **0.0101** | 0.0625 |
| **Arginine** | 0.0027 | 0.0001 | 4.9453 | 0.0032 | 0.0003 | 8.2488 | 0.0029 | 0.0002 | 7.2642 | 0.0031 | 0.0003 | 8.6701 | **0.0284** | 0.3631 | 0.1509 | 0.6307 |
| **Betaine** | 0.0113 | 0.0013 | 11.8688 | 0.0115 | 0.0004 | 3.7426 | 0.0149 | 0.0019 | 12.6931 | 0.0136 | 0.0009 | 6.7688 | 0.8111 | 0.3350 | **0.0420** | **0.0234** |
| **Glycine** | 0.0037 | 0.0001 | 3.1487 | 0.0045 | 0.0002 | 3.7697 | 0.0031 | 0.0001 | 4.7081 | 0.0032 | 0.0003 | 9.6373 | **0.0009** | 0.6461 | **0.0030** | **0.0025** |
| **Glycerol** | 0.0008 | 0.0001 | 14.0170 | 0.0021 | 0.0005 | 25.1808 | 0.0018 | 0.0007 | 42.0832 | 0.0034 | 0.0017 | 50.4505 | **0.0222** | 0.1991 | 0.1121 | 0.2739 |
| **Threonine** | 0.0011 | 0.0001 | 5.1511 | 0.0008 | 0.0000 | 5.5943 | 0.0012 | 0.0001 | 6.9249 | 0.0009 | 0.0001 | 7.8831 | **0.0006** | **0.0014** | **0.0255** | 0.1053 |
| **Glutathione** | 0.0003 | 0.0000 | 16.6010 | 0.0002 | 0.0000 | 21.8373 | 0.0002 | 0.0001 | 29.0329 | 0.0002 | 0.0001 | 34.1408 | 0.1482 | 0.9309 | 0.2166 | 0.7416 |
| **Glucose** | 0.0007 | 0.0001 | 17.4111 | 0.0007 | 0.0001 | 7.3457 | 0.0008 | 0.0001 | 11.2706 | 0.0006 | 0.0001 | 9.2436 | 0.7941 | 0.0582 | 0.3958 | 0.0996 |
| **Trehalose** | 0.0036 | 0.0002 | 4.2524 | 0.0078 | 0.0005 | 6.8653 | 0.0094 | 0.0008 | 8.3378 | 0.0134 | 0.0011 | 8.5714 | **0.0004** | **0.0038** | **0.0007** | **0.0012** |
| **Allantoin** | 0.0001 | 0.0000 | 33.6568 | 0.0004 | 0.0001 | 26.7021 | 0.0002 | 0.0000 | 22.4329 | 0.0002 | 0.0001 | 42.6955 | **0.0103** | 0.2995 | **0.0089** | 0.1151 |
| **NAD^+^** | 0.0002 | 0.0000 | 17.4066 | 0.0001 | 0.0000 | 18.0332 | 0.0001 | 0.0001 | 54.9221 | 0.0001 | 0.0000 | 26.0302 | 0.3926 | 0.8592 | 0.4892 | 0.8623 |
| **Fumarate** | 0.0001 | 0.0000 | 34.1014 | 0.0000 | 0.0000 | 30.6083 | 0.0000 | 0.0000 | 33.3915 | 0.0001 | 0.0000 | 42.0532 | 0.1972 | 0.2373 | 0.0765 | 0.5966 |
| **Tyrosine** | 0.0006 | 0.0001 | 9.5271 | 0.0005 | 0.0001 | 17.6438 | 0.0003 | 0.0000 | 15.9808 | 0.0003 | 0.0000 | 14.7832 | 0.1992 | 0.9562 | **0.0006** | **0.0221** |
| **Histidine** | 0.0004 | 0.0001 | 17.4136 | 0.0004 | 0.0001 | 31.4076 | 0.0003 | 0.0001 | 24.4316 | 0.0003 | 0.0001 | 30.2032 | 0.8331 | 0.9146 | 0.0887 | 0.2675 |
| **Phenylalanine** | 0.0007 | 0.0001 | 13.1329 | 0.0004 | 0.0001 | 19.2551 | 0.0003 | 0.0000 | 11.8977 | 0.0002 | 0.0000 | 12.5015 | **0.0126** | **0.0220** | **0.0021** | **0.0119** |
| **Tryptophan** | 0.0002 | 0.0000 | 0.8977 | 0.0002 | 0.0000 | 6.7918 | 0.0001 | 0.0000 | 19.5263 | 0.0001 | 0.0000 | 18.7185 | 0.1152 | 0.0755 | **0.0202** | 0.2314 |
| **AMP** | 0.0004 | 0.0001 | 27.1688 | 0.0005 | 0.0003 | 67.2105 | 0.0004 | 0.0003 | 59.6178 | 0.0004 | 0.0004 | 95.1959 | 0.5584 | 0.9633 | 0.6421 | 0.8516 |
